# Role of serine/threonine protein kinase STN7 in the formation of two distinct photosystem I supercomplexes in *Physcomitrium patens*

**DOI:** 10.1093/plphys/kiac294

**Published:** 2022-06-23

**Authors:** Caterina Gerotto, Andrea Trotta, Azfar Ali Bajwa, Tomas Morosinotto, Eva-Mari Aro

**Affiliations:** Department of Life Technologies, Molecular Plant Biology, University of Turku, Turku, FI-20014, Finland; Department of Life and Environmental Sciences, Università Politecnica delle Marche, Ancona, 60131, Italy; Department of Life Technologies, Molecular Plant Biology, University of Turku, Turku, FI-20014, Finland; Institute of Biosciences and Bioresources, National Research Council of Italy, Sesto Fiorentino, 50019, Italy; Department of Life Technologies, Molecular Plant Biology, University of Turku, Turku, FI-20014, Finland; Department of Biology, University of Padova, Padova, 35121, Italy; Department of Life Technologies, Molecular Plant Biology, University of Turku, Turku, FI-20014, Finland

## Abstract

Reversible thylakoid protein phosphorylation provides most flowering plants with dynamic acclimation to short-term changes in environmental light conditions. Here, through generating Serine/Threonine protein kinase 7 (STN7)-depleted mutants in the moss Physcomitrella (*Physcomitrium patens*), we identified phosphorylation targets of STN7 kinase and their roles in short- and long-term acclimation of the moss to changing light conditions. Biochemical and mass spectrometry analyses revealed STN7-dependent phosphorylation of N-terminal Thr in specific Light-Harvesting Complex II (LHCII) trimer subunits (LHCBM2 and LHCBM4/8) and provided evidence that phospho-LHCBM accumulation is responsible for the assembly of two distinct Photosystem I (PSI) supercomplexes (SCs), both of which are largely absent in STN7-depleted mutants. Besides the canonical state transition complex (PSI-LHCI-LHCII), we isolated the larger moss-specific PSI-Large (PSI-LHCI-LHCB9-LHCII) from stroma-exposed thylakoids. Unlike PSI-LHCI-LHCII, PSI-Large did not demonstrate short-term dynamics for balancing the distribution of excitation energy between PSII and PSI. Instead, PSI-Large contributed to a more stable increase in PSI antenna size in Physcomitrella, except under prolonged high irradiance. Additionally, the STN7-depleted mutants revealed altered light-dependent phosphorylation of a monomeric antenna protein, LHCB6, whose phosphorylation displayed a complex regulation by multiple kinases. Collectively, the unique phosphorylation plasticity and dynamics of Physcomitrella monomeric LHCB6 and trimeric LHCBM isoforms, together with the presence of PSI SCs with different antenna sizes and responsiveness to light changes, reflect the evolutionary position of mosses between green algae and vascular plants, yet with clear moss-specific features emphasizing their adaptation to terrestrial low-light environments.

## Teaser

Phosphorylation-dependent formation of photosystem I supercomplexes provides both short- and long-term acclimation of moss photosynthetic apparatus to changing environmental cues.

## Introduction

Conversion of sunlight into chemical energy in oxygen-evolving photosynthetic organisms occurs in two thylakoid-embedded photosystems, photosystems II and I (PSII and PSI). The structure and function of the PSII and PSI core complexes are highly conserved from cyanobacteria to angiosperms while the external light-harvesting system has changed in the course of evolution, from soluble phycobilisomes to thylakoid embedded light-harvesting complexes (LHCs; Allen et al., 2011; Crepin and Caffarri, 2018). The LHC proteins of land plants, binding chlorophyll (Chl) *a* and *b*, comprise the LHCII (LHCB proteins) and LHCI (LHCA subunits) antenna systems mainly associated with PSII and PSI, respectively (Alboresi et al., 2008).

Historically, the LHCII antenna size is known to vary in flowering plants from different light intensities (Bailey et al., 2001; Albanese et al., 2016). Yet, natural environments are highly dynamic and the thylakoid-associated photosynthetic electron transport requires continuous modulation to prevent light-induced oxidative damage. Plant chloroplasts harbor a high number of regulatory proteins and pathways which, similarly to the light-harvesting proteins, have changed during evolution. The moss Physcomitrella, recently re-classified as *Physcomitrium patens* (Rensing et al., 2020), shares elements of its photosynthetic apparatus with both vascular plants and green algae. For instance, Physcomitrella accumulates both plant-type and algal-type proteins essential for activation of nonphotochemical energy quenching (NPQ), PSBS, and LHCSR, respectively (Alboresi et al., 2010; Gerotto et al., 2012). Physcomitrella chloroplasts also accumulate flavodiiron proteins (FDP or FLV) to protect PSI upon abrupt exposure to high light, the FDPs being conserved in oxygen-evolving photosynthetic organisms from cyanobacteria to all plants except angiosperms (Zhang et al., 2009; Gerotto et al., 2016; Ilík et al., 2017).

Thylakoid protein phosphorylations likewise play a seminal role to optimize photosynthesis in a variable environment. The light-dependent phosphorylation of LHCII is a regulatory mechanism of thylakoid electron flow in plant chloroplasts to adjust equal distribution of excitation energy to PSII and PSI. Such modulation is based on Serine/Threonine protein kinase 7 (STN7) that phosphorylates the N-terminal Thr residue in specific LHCII trimer subunits [LHCB1 and LHCB2 in Arabidopsis (*Arabidopsis thaliana*)] (Crepin and Caffarri, 2018). Upon phosphorylation, LHCII trimer binds to PSI to enhance energy distribution for PSI, both in light intensity- and light quality-dependent manner (Bellafiore et al., 2005; Grieco et al., 2015, Pan et al., 2018). Conversely, dephosphorylation of LHCII proteins by thylakoid-associated phosphatase TAP38/PPH1 (Pribil et al., 2010; Shapiguzov et al., 2010) leads to a release of LHCII trimer from PSI. Additionally, the STN8 kinase/PSII core phosphatase system preferentially functions in reversible phosphorylation of PSII core proteins D1, D2, CP43, and PsbH in flowering plants to facilitate PSII repair after light-induced damage (Bonardi et al., 2005; Tikkanen et al., 2008; Fristedt et al., 2009; Rochaix et al., 2012; Samol et al., 2012). Although both green algae and nonvascular plants have kinases and phosphatases homologous to those in angiosperms (Depège et al., 2003; Grouneva et al., 2013), there are distinct differences in the specificity, extent, and physiological meaning of thylakoid protein phosphorylations between the different groups (Allorent et al., 2013; Betterle et al., 2015; Grieco et al., 2016). Model species from green algae and angiosperms have been extensively investigated, whereas our knowledge about the role and phosphorylation targets of thylakoid proteins in other land plants, like mosses, liverworts, ferns, or conifers, is only emerging (Ferroni et al., 2014; Grebe et al., 2020). Recent research on Physcomitrella wild-type (WT) and STN8-depleted mutant (Gerotto et al., 2019) revealed no dynamic response of PSII core protein (D1, D2, and CP43) phosphorylation to increasing light intensity, in sharp contrast to flowering plants (Rintamäki et al., 1997). Moreover, the D1 protein appeared not even to be a target for phosphorylation in Physcomitrella, and similar conclusions have been drawn from experiments with a lycophyte, *Selaginella martensii* (Ferroni et al., 2014).

Previous experiments have revealed two main targets of dynamic phosphorylation among LHCII antennae in Physcomitrella in response to white light changes: (1) the trimeric LHCII subunits (Gerotto et al., 2019), which all classified as LHCBM by lacking the clear sequence markers for classification as LHCB1 or LHCB2 as in Arabidopsis (Crepin and Caffarri, 2018); (2) the monomeric antenna LHCB6, for which a specific light-induced dynamic phosphorylation, not detected in Arabidopsis, has instead been revealed for Physcomitrella (Gerotto et al., 2019) as well as for Selaginella (Ferroni et al., 2014). The knowledge on Physcomitrella LHCBM and LHCB6 phosphorylation, however, is still scarce both concerning the kinase(s) responsible for their phosphorylation and the physiological relevance of phospho-LHCBM and phospho-LHCB6 accumulation. In this work, we addressed the STN7 kinase-dependent phosphorylations of Physcomitrella LHCII proteins, the interactions of such phospho-proteins with thylakoid protein complexes, particularly with a distinct PSI-Large complex (Iwai et al., 2018; Pinnola et al., 2018; Gerotto et al., 2019), and their physiological roles in enabling the light acclimation of the moss to changing irradiance conditions.

## Results

### Generation of Physcomitrella STN7 kinase depleted mutants

In difference from model species for angiosperms and green algae possessing a single *STN7* gene (Depège et al., 2003; Bellafiore et al., 2005), the genome of the moss Physcomitrella harbors two genes (*STN7.1*, Pp3c4_25980; *STN7.2*, Pp3c26_5140) encoding a protein homologous to STN7 kinase (Grouneva et al., 2013). These two STN7 isoforms show 83% identity in their amino acid sequences, while the similarity with Arabidopsis sequence is 74% for STN7.1 and 73% for STN7.2 (Supplemental Figure S1). In Arabidopsis, the STN7 kinase is itself a phospho-protein, characterized by three phospho-sites in the C-terminus: Ser-526, Thr-537, and Thr-541 (Trotta et al., 2016). In both Physcomitrella STN7 isoforms, a Gln residue substitutes At Ser-526, whereas the two Thr residues are conserved (Supplemental Figure S1) and were both found phosphorylated by mass spectrometry (MS) analysis (Supplemental Figure S2; Supplemental Table S1).

**Figure 1:**
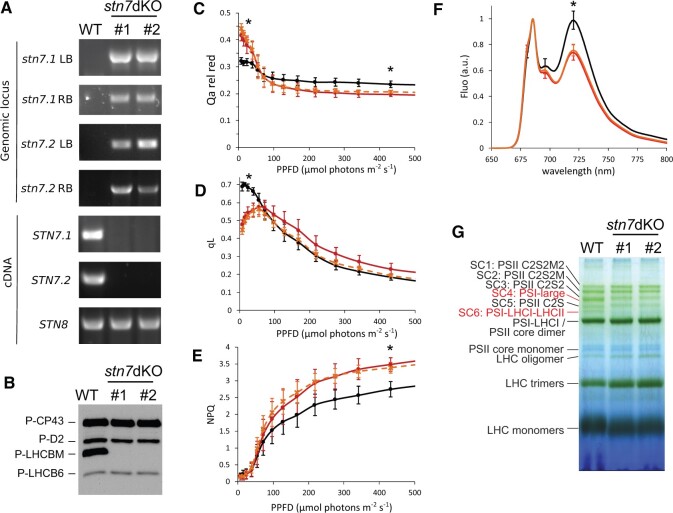
Characterization of Physcomitrella *stn7* double KO lines. A, Insertion of the resistance cassette in *STN7.1* and *STN7.2* gene loci in selected *stn7* double KO lines was verified by amplification of the flanking regions (i.e. Left and Right Borders, LB and RB) of the integrated resistance cassette (*stn7.1* and *stn7.2* LB and RB, on genomic DNA), which proved the insertion occurred at expected position in the genome; amplification of *STN7.1, STN7.2*, and *STN8* transcripts on cDNA, the latter as a positive control, verified the transcription of *STN* genes. The two *stn7* double KO lines (*stn7* dKO #1 and *stn7* dKO #2) were obtained from two independent transformations as described in the text. The characterization of the starting single KO is shown in Supplemental Figure S3. Supplemental Table S2 lists the primers used in the different PCRs. B, anti-P-Thr immunodetection on thylakoid extracts from WT and two *stn7* double KO clones grown in control conditions (CL). About 1 µg of Chl was loaded in each lane. C–E, Qa relative reduction (Qa rel red; C), qL (D) and NPQ (E) parameters from Light curve kinetics shown as average ± SD for WT (black, *n* = 6), *stn7* double KO line 1 (red; *n* = 5) and *stn7* double KO line 2 (dashed orange, *n* = 5). Only the first part of the light curve, from dark to about 500 µmol photons m^−2^ s^−1^, is shown in the figure. The complete charts and additional parameters are depicted in Supplemental Figure S4. Actinic lights of 24, 97, and 430 µmol photons m^−2^ s^−1^ were selected as a proxy for low, moderate, and high illumination. Asterisks indicate when the WT performance statistically differs from that of both *stn7* double KO lines (ANOVA, *P* < 0.05 for Qa relative reduction at 24 µmol photons m^−2^ s^−1^, *P* < 0.001 for Qa relative reduction and NPQ at 430 µmol photons m^−2^ s^−1^, qL at 24 µmol photons m^−2^ s^−1^). F, 77K fluorescence emission spectra of thylakoids from WT (black) and *stn7* double KO lines 1(red) and 2 (orange). The spectra are normalized to the emission peak at 685 nm and are shown as an average of six experiments (with thylakoids extracted from three independent biological replicates); SD is also shown for the peaks of emission. The emission peaks at 720 nm (PSI) are statistically different between WT and both the *stn7* double KO lines (ANOVA, *P* < 0.001). G, lpBN-PAGE of the same samples as (B). Samples were solubilized with 1% β-DM at a final Chl concentration of 0.5 µg/µL. A total of 6 µg of Chl was loaded in each well. The main photosynthetic complexes, as identified in the previous work (Gerotto et al., 2019), are indicated. Red labels highlight the bands that are different in *stn7* double KO compared to the WT. LHC: light-harvesting complexes; PSI (II): PSI (II); PSII C2S(2): PSII SC including a core dimer (C2) and one (S) or two (S2) strongly bound LHCII trimers; PSII C2S2M(2): PSII SC including one (M) or two (M2) moderately bound LHCII trimers additional to C2S2; PSI-Large: PSI SC including PSI core, LHCI, LHCB9, and LHCII antenna isoforms.

To identify the substrates and investigate the physiological role(s) of STN7 kinase(s) in Physcomitrella, we generated mutant lines depleted in STN7.1 only (*stn7.1* single knock-out [KO]), in STN7.2 only (*stn7.2* single KO), or in both STN7.1 and STN7.2 kinase isoforms (*stn7.1/7.2* double KO lines, which will hereafter be referred as *stn7* double KO). Noteworthy, the two *stn7* double KO characterized here were obtained starting either from *stn7.1* single KO or from *stn7.2* single KO lines used as genetic background to subsequently KO the second isoform, generating fully independent double KO lines depleted in both STN7.1 and STN7.2 (Figure 1A; Supplemental Figure S3). After verifying that homologous recombination indeed drove the insertion of the resistance cassette within *STN7.1* and/or *STN7.2* gene locus, and that the respective transcripts were missing (Figure 1A; Supplemental Figure S3; Supplemental Table S2 for primers list), the lack of accumulation of STN7 kinase in the *stn7* double KO was also verified by MS. Several unique peptides for both STN7.1 and STN7.2 were detected in WT sample but were missing in the *stn7* double KO lines (Supplemental Table S3). Conversely, STN8 unique peptides were retrieved in all samples (Supplemental Table S3).

### Phenotype of stn7 double KO mutants of Physcomitrella

In mosses grown in control light conditions (45 µmol photons m^−2^ s^−1^, hereafter, CL), the anti-P-Thr antibody detected the phosphorylation of CP43, D2, and LHCB6 proteins in the thylakoid extracts from all analyzed genotypes, that is, WT, *stn7.1* single KO, *stn7.2* single KO and *stn7* double KO (Figure 1B; Supplemental Figure S3). LHCBM was strongly phosphorylated in WT and single KO lines but was instead not detectable in *stn7* double KO (Figure 1B; Supplemental Figure S3). This suggested that, at least in the growth conditions tested here, one isoform of STN7 kinase is enough to display the WT phenotype in the single KO mutants and thus compensates for the activity of the other. Yet, this result does not exclude a possibility that the two copies of *STN7* gene might have a different expression pattern in specific environmental conditions not analyzed here, like different temperatures, as found previously for the two Physcomitrella LHCSR isoforms (LHCSR1 and LHCSR2; Gerotto et al., 2011). Because of apparent functional overlapping of the two STN7 isoforms, we focused our work only on the *stn7* double KO lines, totally depleted in STN7 kinase accumulation (Supplemental Table S3).

CL-grown *stn*7 double KO lines showed a slightly lower maximum PSII quantum yield than the WT (as revealed by Fv/Fm, Supplemental Figure S4). Light curve experiments demonstrated higher relative Q_A_ reduction (estimated as F/Fm) in *stn7* double KO with respect to WT at low actinic light intensities (Figure 1C), indicating in *stn7* double KO an over-reduction of the plastoquinone (PQ) pool upon exposure to low irradiance. Consistently, *stn7* double KO showed higher PSII saturation, as revealed both by the photochemical quenching (qL; Figure 1D) and Y(II) (Supplemental Figure S4) parameters. These differences disappeared for actinic light intensities >60 µmol photons m^−2^ s^−1^, and >250 µmol photons m^−2^ s^−1^ the *stn7* double KO displayed lower relative reduction of Q_A_ together with a higher NPQ capacity in comparison to WT (Figure 1C–E; Supplemental Figure S4). In contrast to the PSII parameters, the PSI quantum yield of WT and *stn7* double KO lines was undistinguishable (Supplemental Figure S4), indicating that PSI activity was not altered in STN7-depleted mutants.

Next, the *stn7* double KO mosses were tested for the ability to undergo state transitions, that is, to balance the energy distribution between PSII and PSI, a process usually mediated by LHCII phosphorylation in plants (Bellafiore et al., 2005; Betterle et al., 2015). State 2 and State 1 transitions were induced by illuminating the samples with blue light (BL, as state 2 light) and far-red (FR) light (as state 1 light), respectively. In the BL phase, preferentially exciting PSII, WT exhibited strong time-dependent fluorescence quenching, while the *stn7* double KO maintained higher levels of fluorescence, reminiscent of a higher PQ pool reduction, all through the BL phase (Supplemental Figure S5). This resembles the behavior observed in Arabidopsis and rice (*Oryza sativa*) STN7-depleted mutants, suggesting similar molecular mechanism of state transitions (Bellafiore et al., 2005; Pesaresi et al., 2011; Betterle et al., 2015).

The 77K fluorescence emission spectrum of CL thylakoid extracts was characterized by two peaks with maxima at 685 and 696 nm, related to PSII, and third one at 720 nm, related to PSI (Figure 1F; Carbonera et al., 2012; Pinnola et al., 2015). A significantly lower PSI fluorescence emission (720 nm) was observed in *stn7* double KO compared to WT (Figure 1F).

For characterization of chloroplast pigment-binding protein complexes, the thylakoids isolated from Physcomitrella WT and *stn7* mutant lines were solubilized with a mild detergent, n-Dodecyl-β-D-maltoside (β-DM), and subjected to separation by large pore-Blue Native Polyacrylamide Electrophoresis (lpBN-PAGE). The identity of each green band in Physcomitrella WT has been previously verified by generating an MS-based 2D map of the subunits composing each green band (Gerotto et al., 2019). The lpBN band pattern showed that the two PSI supercomplexes (SCs), namely (1) the PSI-Large (SC4), a Physcomitrella-specific PSI SC including LHCB9 and other LHCII antennae subunits (PSI-LHCI-LHCB9-LHCII; Iwai et al., 2018; Pinnola et al., 2018; Gerotto et al., 2019), and (2) the PSI-LHCI-LHCII (also known as “state transition band,” SC6) were almost missing from the *stn7* double KO lines but clearly present in all other genotypes under investigation, that is, WT, *stn7.1* KO and *stn7.2* KO single KO mutants (Figure 1G; Supplemental Figure S3C). Conversely, all the other green bands, that is, PSII SCs (SC1–3 and SC5), PSI-LHCI, PSII core dimer and monomer, and LHCII antenna complexes, were similarly present in all the genotypes analyzed (Figure 1G; Supplemental Figure S3C).

### Thylakoid proteins as targets of the STN7 kinase in Physcomitrella

**Figure 2:**
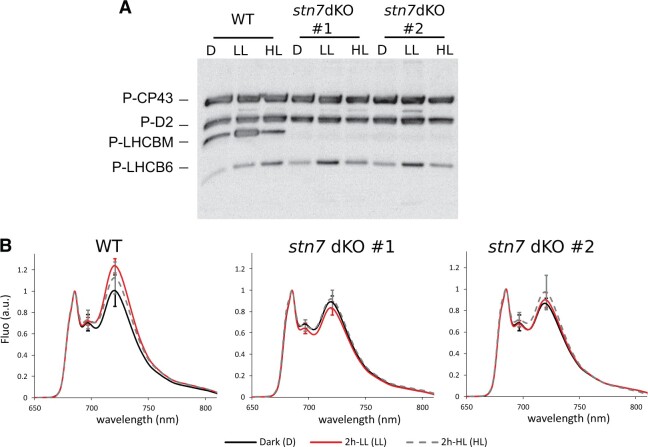
Phenotype of *stn7* double KO upon short-term white light changes (2h-LL and 2h-HL treatments). A, Immunoblotting with anti-P-Thr antibodies of thylakoid extracts from wild-type (WT) and the two independent *stn7* double KO lines (*stn7* dKO #1 and #2). Mosses grown in CL were dark acclimated overnight (D), then exposed for 2h-LL (7 µmol photons m^−2^ s^−1^, LL) and subsequently for 2h-HL (500 µmol photons m^−2^ s^−1^, HL) prior to thylakoid extraction. About 1 µg of Chl was loaded in each lane. The identification of phospho-protein bands is according to Gerotto et al. (2019). Gel electrophoresis experiments were verified with three independent comparisons of the two genotypes. B, 77K fluorescence emission spectra of the WT and *stn7* double KO samples treated as in (A), normalized to their emission at 685 nm. Overnight dark acclimated samples are shown in black, 2h-LL exposed sample in red, and the subsequent treatment with 2h-HL in dashed gray, as shown in the legend below the graphs. Fluo charts are depicted as the average of six experiments with samples from three independent biological replicates. For the main emission peaks at 696 nm and at 720 nm, SD is also shown. WT LL sample peak at 720 nm is significantly different from both the stn7 double KO lines 1 and 2 exposed to 2h-LL (LL) (ANOVA, *P* < 0.001) while no differences were found among dark (D) and HL samples.

We next analyzed the dynamics of antenna protein phosphorylations upon short-term changes in illumination to disclose rapid thylakoid regulation mechanisms. To this end, WT moss cultures grown in CL, after overnight dark acclimation, were exposed for 2 h to different light qualities or intensities to reveal associated changes in thylakoid protein phosphorylation (Supplemental Figure S6). Illumination with red light wavelengths (either 660 or 630 nm) enhanced LHCBM phosphorylation compared to dark-acclimated state, whereas FR light (735 nm) led to almost complete LHCBM dephosphorylation (Supplemental Figure S6, A–B). Illumination with white light of different intensities led to similar effects. A shift from dark to low (2h-LL) and then to high (2h-HL) white light irradiance caused drastic Thr phosphorylation and dephosphorylation of LHCBM, respectively (Supplemental Figure S6C; Gerotto et al., 2019). Moreover, irrespective of the previous acclimation of mosses to darkness or HL, the subsequent LL treatment always induced the strongest LHCBM phosphorylation level in WT (Supplemental Figure S6C). In general, the changes in LHCBM phosphorylation by any light treatment were reflected in the relative intensity of 77K PSI fluorescence emission peak at 720 nm (Supplemental Figure S6D). In particular, a clear 2h-LL-induced increase in fluorescence emission from PSI (peak at 720 nm) was visible in WT, whilst in *stn7* double KO, where LHCBM Thr-phosphorylation is missing (Figure 2A), the 77K spectra remained highly similar independently of the different light treatments (Figure 2B).

Intriguingly, the monomeric antenna LHCB6 revealed unique phosphorylation dynamics in Physcomitrella. Treatment of WT with red light (630 and 660 nm; Supplemental Figure S6), and with stepwise increasing intensity of white light from darkness to HL (Figure 2A) enhanced LHCB6 phosphorylation. Although HL-induced LHCB6 phosphorylation was not dependent on STN7 (Gerotto et al., 2019), in *stn7* double KO it was altered and the highest level of LHCB6 phosphorylation was occurring in LL and decreased in HL (Figure 2A; Supplemental Figure S7).

### Short-term dynamics of thylakoid protein complexes in Physcomitrella stn7 double KO

**Figure 3:**
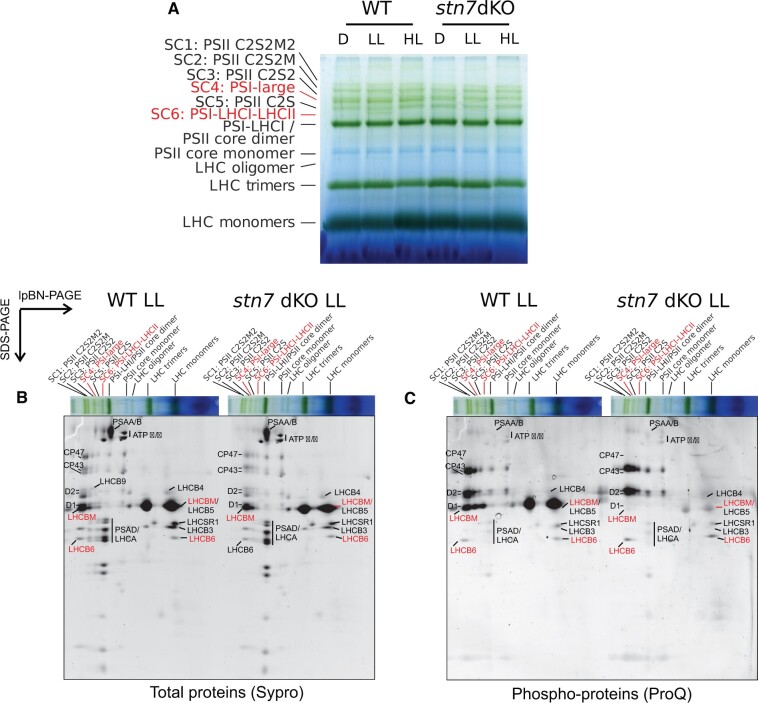
2D-lpBN-SDS-PAGE total and phospho-protein maps of WT and *stn7* double KO thylakoids. A, lpBN-PAGE on thylakoid extracts of wild-type (WT) and *stn7* double KO dark acclimated overnight (D), then exposed for 2 h to LL (7 µmol photons m^−2^ s^−1^, LL) and subsequently for 2h-HL (500 µmol photons m^−2^ s^−1^, HL) prior to thylakoid extraction, solubilization with 1% β-DM at a final Chl concentration of 0.5 µg/µL and the separation of protein complexes in lpBN-PAGE. A total of 6 µg of Chl was loaded in each lane. *stn7* double KO line #1 (*stn7* dKO) is depicted in the figure, but similar results were obtained with line #2. B–C, 2D-lpBN-SDS-PAGE on 2h-LL treated mosses (WT and *stn7* double KO line #2 are shown, but similar results were obtained with *stn7* double KO line #1), stained with Sypro Ruby, showing total proteins (B), and with the phospho-protein specific dye ProQ Diamond (C). Green bands and phospho-proteins which showed differential accumulation in WT and *stn7* double KO are highlighted with red labels. The main subunits of PSII and PSI are also indicated in the 2D map. Green bands and spots are named according to (Gerotto et al., 2019). 2D-lpBN-SDS-PAGE of starting dark acclimated and 2h-HL samples are shown in Supplemental Figure S8. Gel electrophoresis experiments were verified with three independent replicates each genotype.

For more in-depth analysis of the short-term dynamics of thylakoid protein complexes, the thylakoid samples isolated from dark acclimated, 2h-LL and 2h-HL exposed WT and *stn7* double KO plants were subjected to separation by nondenaturing lpBN-PAGE. The green band identified as PSI-Large (SC4) was present in all differentially light-treated WT mosses, but nearly missing from all the *stn7* double KO samples (Figure 3A). In addition, upon 2h-LL exposure, the PSI-LHCI-LHCII (SC6) complex accumulated in WT but not in the STN7-depleted mutants (Figure 3A).

The subsequent analyses of 2h-LL thylakoid samples by 2D-lpBN-SDS-PAGE confirmed the presence of three PSI complexes in the WT (PSI-Large, PSI-LHCI-LHCII and PSI-LHCI), while in *stn7* double KO only very scarce signals of PSI-Large or PSI-LHCI-LHCII related subunits were found (Figure 3B). Instead, no distinct differences between the two genotypes were evident among the other main protein complexes, with similar subunit intensity originating from PSI-LHCI subunits, PSII core (CP47, CP43, and D2) and LHCB antennae (Figure 3B).

The same 2D-lpBN-SDS-PAGE was also stained with ProQ dye to detect proteins phosphorylated at Thr, Ser or Tyr residues and to identify which of the green bands included phosphorylated subunits (Figure 3C). A different pattern of phospho-proteins in WT and the *stn7* double KO was clearly visible. In WT, strong phospho-LHCBM signals were detectable in all PSI and PSII SCs, LHCII trimers and LHCII monomers, whereas the phosphorylation signals from these bands in the *stn7* double KO were weak and apparently due to residual phosphorylation on Ser residues (Supplemental Table S1). Notably, among the phospho-peptides detected in the monomeric LHCBM spot of both WT and *stn7* double KO, an N-terminal peptide common to LHCBM3/6/9/10 isoforms was found to be simultaneously phosphorylated on two Ser residues (Supplemental Table S1). The phospho-signals originating from LHCB6 spots, either as a free protein or within PSII SC1-2, were stronger in the *stn7* double KO than in WT, in line with enhanced LHCB6 phosphorylation observed with anti-P-Thr detection (Figures 2A and 3C).

The 2D-lpBN-SDS-PAGE analyses of the 2h-HL thylakoids showed in WT a unique phospho-LHCBM spot among the SCs, which co-localized with PSI-Large subunits (SC4, red asterisks in Supplemental Figure S8). The PSII SCs were free from phosphorylated LHCBM, although the bigger ones C2S2M(2) still included phospho-LHCB6, consistent with the changes detected with anti-P-Thr.

### Long-term light acclimation of Physcomitrella WT and stn7 double KO mutant

**Figure 4:**
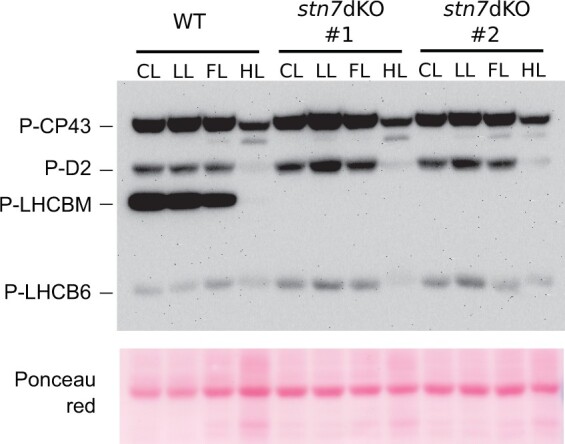
Thylakoid protein phosphorylation upon long-term acclimation to different light regimes. anti-P-Thr immunoblotting on thylakoid extracts of wild-type (WT) and the two independent *stn7* double KO lines (*stn7* dKO #1 and #2) after 7 d of acclimation to long-LL (LL), long-FL (FL) or long-HL (HL) (7, 25/800, or 600 µmol photons m^−2^ s^−1^, respectively, see “Materials and methods” section for further detail on acclimation protocol). Thylakoids from control conditions grown samples (CL) are also included. About 1 µg of Chl was loaded in each lane. Ponceau red staining is shown as a loading control. Gel electrophoresis experiments were verified with three independent comparisons of the two genotypes.

In addition to the standard growth light conditions (CL), mosses were also grown for a week in low (long-LL), fluctuating (long-FL) or high (long-HL) irradiance, with attention being paid particularly on changes in the photosynthetic apparatus induced by long-term exposure of the moss to the different light regimes. First, we checked whether the WT pattern of the lpBN bands was affected by acclimation to long-LL, long-FL, and long-HL (Supplemental Figure S9). WT mosses acclimated to CL and long-LL showed fairly similar pattern, while in long-FL the PSI-LHCI-LHCII band was weak. Nevertheless, the PSI-Large was present in CL, long-LL, and long-FL, conversely to the results in long-HL where both PSI SCs, as well as the PSII SCs, were hardly detectable. It was next analyzed whether these differences were reflected in phosphorylation of thylakoid proteins (Figure 4). In the WT, long-LL, CL, and long-FL revealed roughly similar phosphorylation levels, yet with a slight decrease in LHCBM phosphorylation in the long-FL. In contrast, long-HL acclimation induced a strong decrease in phosphorylation levels of all phospho-proteins detected by anti-P-Thr, including LHCBM in comparison to other long-term light conditions. This behavior was common also for long-HL acclimation of the *stn7* double KO mutants which, besides lacking, as expected, the phosphorylation of LHCBM in all conditions, additionally demonstrated the strongest phosphorylation of LHCB6 in long-LL.

With respect to the 77K emission spectra, the long-HL acclimated WT and *stn7* double KO mutants showed practically no difference, yet the PSI emission peak was substantially reduced in relation to the PSII peak in all long-HL samples in comparison to that of WT under CL (Supplemental Figure S10), in line with the negligible phosphorylation of LHCBM also in the WT. On the other hand, after long-LL, the PSII maximum quantum yield was slightly lower in *stn7* double KO than in the WT (Supplemental Figure S11), whereas the differences induced by long-FL between WT and *stn7* double KO were less obvious. Long-HL instead was equally detrimental for PSII photochemical efficiency in both genotypes (Supplemental Figure S11).

The chl *a/b* ratio, however, was rather constant in the different WT samples but was lower in *stn7* double KO with respect to WT in CL, long-LL and long-FL (Supplemental Table S4).

### Unique features of PSI-Large

**Figure 5:**
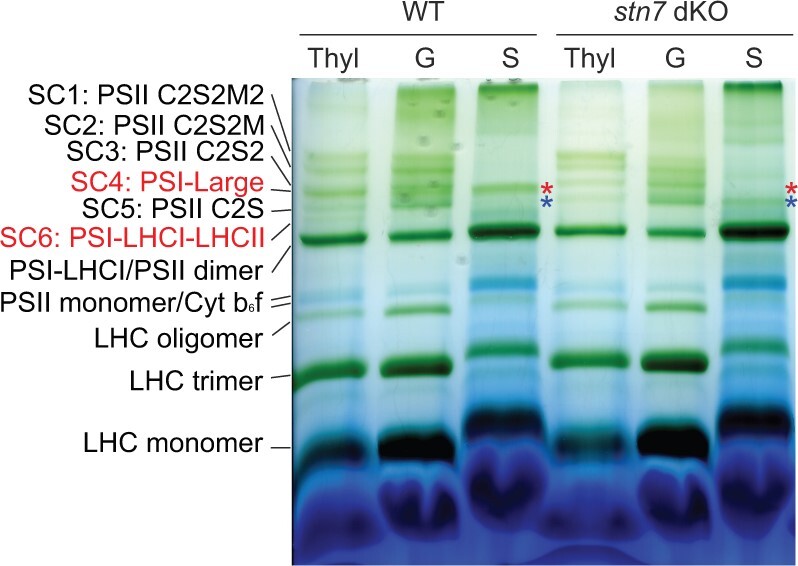
Thylakoid fractionation and isolation of PSI-Large. lpBN-PAGE of whole thylakoids (Thyl), grana (G), and stroma lamellae (S) fractions from wild-type (WT) and *stn7* double KO (*stn7* dKO) samples. Mosses treated with long-FL (25/800 µmol photons m^−2^ s^−1^, see “Materials and methods” section for further detail on FL acclimation protocol) were used in the gel shown, but CL-grown samples displayed similar results, the only difference being a stronger PSI-LHCI-LHCII (SC6) band in WT CL as compared to the WT FL shown here, in line with results shown in Supplemental Figure S9. Upper asterisks (red) indicate the PSI-large band in WT and the respective gel region in *stn7* double KO sample, where no green band is detected. Lower asterisks (blue) instead indicate a PSI SC, as characterized afterwards by 2D-lpBN-SDS-PAGE (see Figure 6), unique to *stn7* double KO. Thylakoid samples (Thyl) were solubilized with 1% w/v β-DM at a final Chl concentration of 0.5 µg/µL, while grana (G) and stroma lamellae (S) fractions were obtained as detailed in the “Materials and methods” section and then solubilized as the thylakoids. A total of 6 µg of Chl was loaded in each lane. LHC: light harvesting complexes; PSI(II): PSI (II); PSII C2S(2): PSII SC including a core dimer (C2) and one (S) or two (S2) strongly bound LHCII trimers; PSII C2S2M(2): PSII SC including one (M) or two (M2) moderately bound LHCII trimers additional to C2S2; PSI-Large: PSI SC including PSI core, LHCI, LHCB9, and LHCII antenna isoforms.

To gain more information on the role of STN7 in the formation of Physcomitrella-specific PSI-Large complex, thylakoids were isolated from WT and *stn7* double KO and fractionated into stroma-exposed and grana membranes. Thylakoids from long-FL acclimated mosses were chosen as they accumulated PSI-Large (SC4) as CL-grown sample, but the accumulation of PSI-LHCI-LHCII was low (SC6; Figure 5; Supplemental Figure S9), decreasing possible contaminations from other PS SCs during PSI-Large characterization.

**Figure 6:**
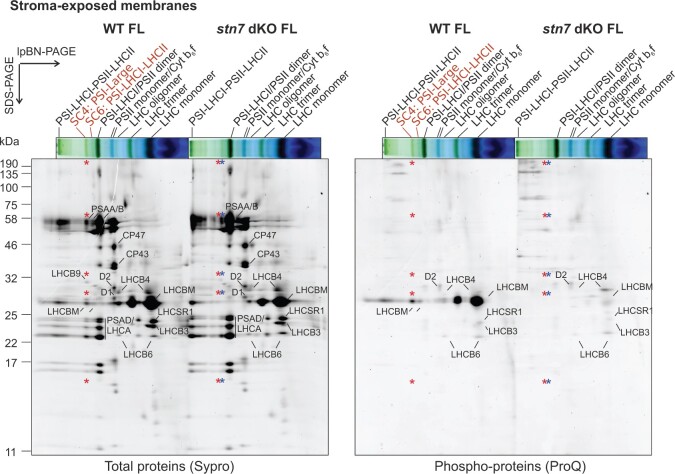
Characterization of stroma lamellae fraction by 2D-lpBN-SDS-PAGE. 2D-lpBN-SDS-PAGE of stroma lamellae-enriched fraction from long-FL (Fluctuating Light, 25/800 µmol photons m^−2^ s^−1^, see “Materials and methods” section for further detail on FL acclimation protocol) thylakoids from wild-type (WT) and *stn7* double KO (*stn7* dKO) was stained with Sypro Ruby, showing the total proteins (left panel), and with the phospho-protein specific dye ProQ Diamond (right panel). 2D-lpBN-SDS-PAGE profiles of the whole thylakoids and the grana-enriched fraction from the same experiment are depicted in Supplemental Figure S12. Green bands which showed a different accumulation in WT and *stn7* double KO are highlighted with red labels. The main subunits of PSII and PSI are also indicated in the 2D map. Green bands and spots are named according to Gerotto et al. (2019), the asterisks indicate the same protein complexes marked in Figure 5. LHC: light harvesting complexes; PSI (II): PSI (II); PSII C2S(2): PSII SC including a core dimer (C2) and one (S) or two (S2) strongly bound LHCII trimers; PSII C2S2M(2): PSII SC including one (M) or two (M2) moderately bound LHCII trimers additional to C2S2; PSI-Large: PSI SC including PSI core, LHCI, LHCB9, and LHCII antenna isoforms.

The analysis of thylakoid fractions by native lpBN-PAGE and 2D-lpBN-SDS-PAGE indicated the grana fractions of both genotypes to be enriched in PSII SCs, as expected (Figure 5; Supplemental Figure S12). PSI-Large was instead detected mostly in the stroma-exposed membranes of WT, as indicated by well-resolved PSI core and LHCA subunits, LHCB9 and phospho-LHCBM spot, but missing in the *stn7* double KO samples (Figures 5 and 6, red asterisks; Supplemental Figure S12).

In the WT stroma-exposed membrane fraction, subunit analysis and staining with ProQ revealed a well-defined phosphorylated LHCBM spot in PSI-Large (Figure 6), while no visible co-migrating PSII subunits were detected. In the stroma-exposed membranes of *stn7* double KO, the PSI-Large band was missing (Figure 5, red asterisk) but a weak band from another PSI SC of a smaller size than the WT PSI-Large and lacking the (phosphorylated) LHCBM subunits was visible (Figures 5 and 6, blue asterisks), which might represent the PSI SC scaffold on which phospho-LHCBM trimer binds to form PSI-Large.

ProQ staining reveals indistinctively the phosphorylation of Ser, Thr or Tyr (Figure 6). Anti-P-Thr immunoblotting of PSI-Large and other SC bands excised from lpBN specifically revealed the Thr-phosphorylation for LHCBM in both PSI-Large and PSI-LHCI-LHCII (Supplemental Figure S13). Noteworthy, in parallel to the missing detection of PSII subunits co-migrating with PSI-Large in Figure 6, no signals of phospho-CP43 and phospho-D2 were detected in the PSI-Large band (Supplemental Figure S13), indicating the PSI-Large complex from stroma lamellae fraction, used in these analyses, was not contaminated by PSII SCs, thus assuring that the phosphorylated LHCBM band was specifically bound to PSI.

### Identification of LHCBM and LHCB6 phospho-sites

**Figure 7:**
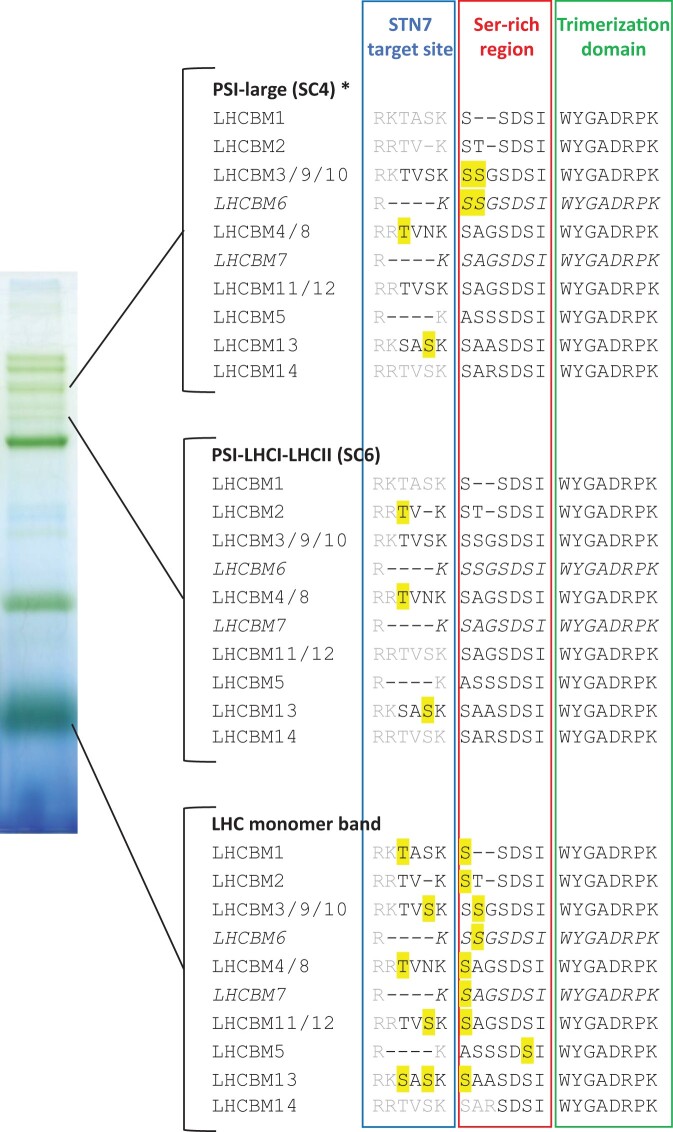
Phosphorylation detected in LHCBM isoforms. Schematic view of LHCBM phospho-sites detected in solubilized LHC monomers, PSI-LHCI-LHCII (state transition band, SC6) and PSI-Large (SC4). Letters indicate the N-terminus sequence of the different LHCBM isoforms, grouped when the sequences are identical, that is, when they originated the same peptide after trypsin digestion and cannot be singularly identified. Note that LHCBM6 and LHCBM7 are reported in italics to emphasize they share only a part of their N-terminal sequence with LHCBM3/9/10 and LHCBM4/8/11/12, respectively. Black letters indicate the residues found in MS-detected peptides, as opposed to gray letters that were not detected by MS. Phosphorylated residues detected by MS, as reported in Supplemental Table S1, are highlighted in yellow. Green bands used for MS analyses were obtained from samples grown in different conditions (CL for LHC monomers, CL and long-FL for PSI-Large, long-LL for PSI-LHCI-LHCII). Asterisk for PSI-Large indicates the band was retrieved from stroma lamellae fraction, to avoid contamination from nearby PSII SCs, while PSI-LHCI-LHCII and LHC monomer bands were cut from whole thylakoid lpBN, like from the representative lpBN lane from whole thylakoids shown here as a visual reference for lpBN bands analyzed.

In vascular plants, the trimeric LHCII main subunits can be distinguished as LHCB1, LHCB2, and LHCB3 (Koziol et al., 2007; Crepin and Caffarri, 2018; Grebe et al., 2019). Conversely, in green algae as well as in Physcomitrella, the specific sequence features of LHCB1 and LHCB2 are not identifiable, and, besides LHCB3, the different LHCII isoforms are all assigned as LHCBM (Alboresi et al., 2008; Crepin and Caffarri, 2018). Still, Physcomitrella LHCBM sequences can be grouped according to specific features of their N-terminus (Figure 7; Supplemental Figure S14). Most of them include a sequence similar (LHCBM1/4/8-11/12-14) or even identical (LHCBM2) to the “STN7 target site,” identified in Arabidopsis as the sequence R(R/K)TV(K/R) (Crepin and Caffarri, 2018). LHCBM13 harbors a Ser in place of a Thr (Figure 7). LHCBM5, LHCBM6, and LHCBM7 present a shorter N-terminus, which lacks a Thr residue after the N-terminal positively charged amino acids required for STN7 recognition (RR of RK; Liu et al., 2016; Figure 7; Supplemental Figure S14). Instead, LHCBM5, LHCBM6, and LHCBM7, as most of the Physcomitrella LHCBM, are characterized by a region enriched in Ser residues (which we called “Ser-rich” region) not conserved in Arabidopsis LHCB1 and LHCB2 (Figure 7; Supplemental Figure S14).

The N-terminus sequences of LHCBM proteins thus include several Thr/Ser residues potentially prone to phosphorylation. Searching for the phosphorylated N-terminal Thr responsible for the anti-P-Thr signal in immunoblotting, we used the abundant LHCBM monomer band from native lpBN-PAGE as a starting material for MS analyses. Many N-terminus peptides were detected, some as phospho-peptides. The N-terminal Thr phosphorylation of LHCBM4/8 was found (Figure 7; Supplemental Table S1). Moreover, LHCBM3/9/10, LHCBM11/12, and LHCBM13 were found phosphorylated on the Ser within the STN7 target site. In the case of LHCBM1, the phosphorylated residue of STN7 target site could not be determined unambiguously (Thr or Ser) (Figure 7; Supplemental Table S1). Many other Ser residues of the N-terminus were also found phosphorylated within the “Ser-rich region.”

The same analyses on the PSI-LHCI-LHCII band revealed phospho-Thr in LHCBM2 and LHCBM4/8 N-terminus, while LHCBM13 was phosphorylated at N-terminal Ser (Figure 7; Supplemental Table S1). PSI-Large complex, excised from the lpBN gel of stroma-exposed membrane fraction to maximally avoid the contaminations by co-migrating complexes, revealed as well the N-terminal phosphorylations of LHCBM subunits. In particular, the N-terminal Thr of LHCBM4/8, along with LHCBM13 N-terminal Ser, were found phosphorylated, together with a doubly phospho-Ser peptide in the Ser-rich region shared by LHCBM3/6/9/10. Notably, while we directly confirmed the above-mentioned phospho-sites indicating that both PSI-Large and PSI-LHCI-LHCII in Physcomitrella include the N-terminal Thr phosphorylated LHCBM subunits, we cannot fully exclude that other N-terminal phosphorylations are likewise present but were not found in these samples due to the low amount of starting material, and thus to a detection limit for analysis of PSI-LHCI-LHCII and PSI-Large.

As to the phospho-sites of LHCB6, in addition to those previously reported (Gerotto et al., 2019), we here detected the peptide ATKKVSARPAAGGK phosphorylated on Thr-2 (Thr-48 on the complete protein sequence including the transit peptide; Supplemental Table S1). This peptide most likely represents the LHCB6 N-terminus of the mature protein recognized by the anti-P-Thr antibody, indicating that also this antenna can be phosphorylated at the N-terminal Thr in Physcomitrella.

## Discussion

### Phosphorylation dynamics of Physcomitrella LHCII antennae: canonical STN7-dependent LHCBM phosphorylation and the enigmatic LHCB6 phosphorylation

Biochemical and MS analyses revealed that Physcomitrella STN7 kinases specifically target the N-terminal Thr residues of LHCBM protein(s) (Figures 1B, 2A, and 4; Supplemental Table S1), the building blocks of LHCII trimers in mosses. The lack of LHCBM phosphorylation in Physcomitrella *stn7* double KO abolished the light condition-dependent formation of the PSI-LHCI-LHCII complex (state transition complex, SC6) (Figures 1G and 3A). From a functional point of view, this matched with the inability of *stn7* double KO to activate state transitions and balance the energy distribution between PSII and PSI upon short-term changes in irradiance (Figure 1, C–F; Supplemental Figures S3 and S5), in accordance with the canonical function of LHCII trimer phosphorylation in angiosperms (Lunde et al., 2000; Rintamäki et al., 2000; Bellafiore et al., 2005). LHCBM phosphorylation alleviates the reduction pressure on PQ pool and allows optimization of photosynthesis in low light (Figure 1C). Conversely, the *stn7* double KO at higher light irradiance increased NPQ in comparison to WT (Figure 1E). This behavior resembles that reported in Arabidopsis (Hepworth et al., 2021), suggesting that the dephosphorylation of LHCII in HL (Figure 2A) contributes to photoprotection.

In Arabidopsis, LHCB2 (but not LHCB1) is the phosphorylated LHCII trimer subunit interacting with PSI subunit to form the PSI-LHCI-LHCII complex (Crepin and Caffarri, 2015). Arabidopsis LHCB2 isoforms are characterized by the positively charged amino acids “RR” prior to the STN7 target Thr, instead of the pair “RK” found in LHCB1, with such “RR” feature shown to lead to a faster phosphorylation compared to the “RK” sequence of LHCB1 (Liu et al., 2016; Crepin and Caffarri, 2018). Among the Physcomitrella LHCBM bound to PSI-LHCI-LHCII, the LHCBM2, and LHCBM4/8 isoforms were also found as phosphorylated at their N-terminal Thr and they both harbor positively charged amino acids in the form “RR,” like in LHCB2 of Arabidopsis (Figure 7; Supplemental Figure S12). Although in Physcomitrella only LHCBM2 carries an N-terminus identical to that of Arabidopsis LHCB2, the “RR” signature for LHCII phosphorylation and binding to PSI appear to be a conserved feature of state transitions through the evolution of land plants.

Physcomitrella-specific thylakoid protein phosphorylation also displays evolutionarily divergent features, like a dynamic phosphorylation of the monomeric antenna LHCB6. LHCB6 is evolutionarily one of the most recent antenna isoforms, typical of most land plants, while *LHCB6* genes are missing from genomes of green algae and spruce (Crepin and Caffarri, 2018; Grebe et al., 2019). LHCB6 phosphorylation has previously been reported only in the lycophyte Selaginella and was assigned a role in photosynthesis regulation under HL (Ferroni et al., 2014, 2016), while no dynamic phosphorylation has been detected for LHCB6 in angiosperms (Grieco et al., 2015). Multiple aspects of LHCB6 phosphorylation in Physcomitrella still remain enigmatic. Although STN8 kinase was shown to be responsible for the HL-induced LHCB6 phosphorylation (Gerotto et al., 2019), the identity of the kinase responsible for the basal LHCB6 phosphorylation remains unknown. Enhanced phosphorylation of LHCB6 in *stn7* double KO implies that STN7 is not the other kinase targeting LHCB6 (Figures 2 and 4). Physcomitrella genome holds yet another sequence encoding a putative Ser/Thr kinase, with similarity to STN7 and STN8 kinases and predicted localization to chloroplast (Pp3c10_6850V3; Supplemental Figure S1). Nevertheless, the elucidation whether this sequence encodes the “missing” second LHCB6 kinase waits for further research. WT and *stn7* double KO likewise differed with respect to the light condition that induces maximal LHCB6 phosphorylation, being HL and LL, respectively (Figures 2 and 4). Such distinctive behavior hinders the understanding of exact physiological meaning of reversible LHCB6 phosphorylations in Physcomitrella but, on the other hand, suggests a specific function in each light condition, for example, the dissipation of excess light energy in HL and enhancing light harvesting in LL. Such an opposite physiological function of LHCB6 phosphorylation may be acquired by Physcomitrella by employing different kinases and/or by phosphorylation of different/multiple LHCB6 residues identified as phospho-sites (Supplemental Table S1; Gerotto et al., 2019). Complex implementations of antenna protein phosphorylations were previously suggested for *Chlamydomonas reinhardtii* LHCBM proteins, which in phosphorylated form are known to increase the absorption cross-section of PSI but were also proposed to function as a “quenching-type” LHCBM antenna, the latter in stark difference to angiosperms (Allorent et al., 2013; Nagy et al., 2014; Ünlü et al., 2014; Minagawa and Tokutsu, 2015). The extents and specific characteristics of the light-harvesting and energy-quenching modes of LHCBM phosphorylations remain under investigation (Nawrocki et al., 2016). In the PSII-LHCII SC of angiosperms, LHCB6 replaces a position occupied by an LHCBM-trimer in Chlamydomonas (Caffarri et al., 2009; Drop et al., 2014). Whether the complexity of LHCB6 phosphorylation in Physcomitrella provides flexibility for photosynthesis regulation depending on the light intensity, remains still an open question.

### Phosphorylated LHCII trimer is pivotal for assembly of PSI-Large

Intriguingly, beside the PSI-LHCI-LHCII complex, active STN7 kinase appeared to be a prerequisite also for formation of the Physcomitrella bigger PSI SC, the PSI-Large (SC4; Figures 5 and 6), a recently identified PSI SC characterized by a big antenna size (Iwai et al., 2018; Pinnola et al., 2018).

The formation of Physcomitrella PSI-Large seems to rely on complex structural requirements. Previously, the expression of a Physcomitrella-specific antenna protein, LHCB9 (Alboresi et al., 2008), was shown to be a prerequisite for PSI-Large accumulation (Iwai et al., 2015, 2018; Pinnola et al., 2018). LHCB9 is present also in our lpBN green band defined as the PSI-Large (SC4; Figures 3, 5, and 6), as confirmed by MS detection of the protein among the PSI-Large subunits, both from 2D-lpBN-SDS-PAGE spot (Gerotto et al., 2019), and from lpBN green band (Supplemental Table S1). Associated with PSI-Large in SC4, we identified also phosphorylated LHCBM subunits (Figure 6; Supplemental Figure S13). The phospho-LHCBM prevailed in PSI-Large irrespective of the light regime (Figure 3; Supplemental Figures S8, S13, and S14). Indeed, PSI-Large was found in all tested light conditions in which at least a residual LHCBM phosphorylation was detected, even when the accumulation of the other PSI SC, PSI-LHCI-LHCII, was negligible. This is the case with the 2h-HL sample, in which PSI-LHCI-LHCII was almost absent and the residual LHCBM phosphorylation colocalized with the PSI-Large (Supplemental Figure S8). The long-HL condition, instead, nearly depleted LHCBM phosphorylation as well as the PSI-Large complex from the thylakoid membrane (Figure 4; Gerotto et al., 2019), a further confirmation of the link between LHCBM phosphorylation and PSI-Large accumulation in Physcomitrella. Taken together with negligible accumulation of PSI-Large in *stn7* double KOs, it is apparent that LHCBM phosphorylation is pivotal for stable PSI-Large assembly, in addition to LHCB9.

Although PSI-Large assembly clearly relies on LHCBM phosphorylation, like PSI-LHCI-LHCII, the two PSI SCs differ in their dissociation dynamics. In stark contrast to PSI-LHCI-LHCII, the accumulation of PSI-Large, as revealed by the lpBN gel separation, remained almost unaffected after overnight darkness, after 2 h exposure to different white light intensities (Figure 3A), or by long-term acclimation to different light regimes with a prolonged growth in HL as an only exception, a condition that induces PSI-Large disassembly (Supplemental Figure S9; Iwai et al., 2015, 2018; Gerotto et al., 2019). The mechanistic mystery to explain the stability or slow disassembly of PSI-Large upon short-term changes in light conditions, at stark difference from PSI-LHCI-LHCII, remains to be elucidated. Structural analysis of PSI-Large revealed the presence, along with the LHCBM trimer, of additional other 4–5 LHC subunits (suggested to be one LHCB9 and an additional LHCI belt), with some of them being close to the LHCBM trimer (Iwai et al., 2018). It is conceivable that the phosphorylated Thr residues of LHCBM trimer remain protected from de-phosphorylation by the interaction with the “extra” LHCs, thus leading to an almost light intensity-independent accumulation of PSI-Large. However, we cannot exclude that other specific features of Physcomitrella LHCBM proteins might also contribute to PSI-Large assembly and enhanced stability. Our analyses revealed, among PSI-Large subunits, a presence of an LHCBM peptide simultaneously phosphorylated on two Ser residues in the Ser-rich region of Physcomitrella LHCBM3/6/9/10 isoforms (Supplemental Table S1; Figure 7), a region not conserved with Arabidopsis LHCB1/2 sequences (Supplemental Figure S14). Although such Ser phosphorylations are not STN7-dependent (Supplemental Table S1), alike other N-terminal Ser phosphorylation in Arabidopsis LHCB1/2 subunits (Crepin and Caffarri, 2018), the peculiar aminoacidic sequence of LHCBM isoforms, together with their specific multiple phospho-sites, proposes a capability of LHCBM3/6/9/10 N-terminus to mediate unique interactions. Thus, it is conceivable that the Physcomitrella-specific pattern of multiple Ser/Thr phosphorylations within the LHCBM trimer renders the stability of the PSI-Large SC, while the STN7-dependent Thr phosphorylation is essential for the full assembly of PSI-Large.

The physiological relevance of PSI-Large accumulation still needs to be fully elucidated. Several algal species have been recognized to harbor a PSI characterized by a bigger antenna size with respect to plant-type PSI-LHCI, comprising a 4 LHCA belt as a light-harvesting system. Chlamydomonas and other green algae are characterized by 10 LHCA bound to PSI (Ozawa et al., 2018; Van Den Berg et al., 2020; Watanabe and Minagawa, 2020; Huang et al., 2021), the red lineage alga *Nannochloropsis gaditana* is predicted to have at least five LHC subunits in the PSI complex (Alboresi et al., 2017), while the diatom *Chaetoceros gracilis* has up to 24 LHC bound to PSI (Xu et al., 2020). PSI complexes harboring a big antenna size thus appear as a feature of aquatic environments, yet the specific structure and binding site(s) for additional LHCs differ among various algal species.

Physcomitrella PSI-Large provides a further example of “big” PSI SC structures evolved in a nonvascular land plant. The three main PSI complexes, the flowering-plant-like PSI-LHCI and PSI-LHCI-LHCII and the PSI-Large, present in differential relative amounts in the thylakoid membrane and with diversified capabilities to dynamically respond to changing light conditions (Figures 1, 3, 5, and 6; Supplemental Figures S8 and S12), collectively provide Physcomitrella with the means to cope with both low and high irradiances in their natural habitats. The three sizes of PSI in Physcomitrella might be particularly advantageous for life at the land/water interface, as suggested earlier (Iwai et al., 2018), to guarantee maximal PSI activity for maintenance of efficient photosynthesis also under light limiting conditions. Noteworthy, the *stn7* double KO lines are characterized by a higher chronic reduction state of PQ in low/moderate light compared to the WT (Supplemental Figures S4 and S11), indicating that indeed the assembly of PSI SCs contributes to photosynthesis optimization in those light regimes. Such a strategy—the almost constitutive accumulation of a big PSI-Large—is possible for mosses due to the presence of FDPs (missing from angiosperms; Zhang et al., 2009; Ilík et al., 2017), which function as efficient electron acceptors from PSI and thereby provide protection to PSI against photodamage in conditions of a sudden increase in light intensity (Gerotto et al., 2016).

### Conclusions: functional and evolutionary perspectives of the phosphorylation of LHCBM and LHCB6 antenna subunits in Physcomitrella

Thylakoid protein phosphorylation is a strategy plants use to acclimate to short- and long-term fluctuations in irradiance. Reversible phosphorylation of LHCII trimers has been frequently associated with a minute-scale mechanism to balance the energy distribution between PSII and PSI in plants, via the so-called state transitions (Bellafiore et al., 2005; Pesaresi et al., 2011; Betterle et al., 2015). More recently, it has become evident that LHCII trimers serves as an antenna for both PSs also after prolonged illumination under different light regimes (Grieco et al., 2012; Wientjes et al., 2013; Grieco et al., 2015). Alike the model angiosperm Arabidopsis, Physcomitrella displayed short-term dynamics of the PSI-LHCI-LHCII complex (Figure 3), as well as the association of LHCII trimers with both PSs in different growth conditions (Figure 1; Supplemental Figure S9). The light condition-specific behavior of WT and STN7-depleted mosses during acclimation, however, suggests that the actual contribution of STN7 activity to moss photosynthetic acclimation may vary according to the specific light regime applied (Supplemental Figure S11). Further, Physcomitrella light-acclimation strategies appear partly differentiated from that of flowering plants. We present here two Physcomitrella-specific LHCII phosphorylation systems. One, still enigmatic, targeted to the monomeric LHCB6 protein and the other allowing the phospho-LHCBM-dependent accumulation of PSI-Large, in addition to the canonical PSI-LHCI-LHCII. These mechanisms might have developed in early land plants and subsequently disappeared in the course of evolution of flowering plants with more complex plant morphology, paralleled by the appearance of diverse acclimation mechanisms, like the modulation of the size of the light-harvesting antenna upon prolonged exposure to changed light intensity, which instead is negligible in mosses and lycophytes (Aro, 1982; Gerotto et al., 2011; Ferroni et al., 2016; Supplemental Table S4). Progress in understanding the species biodiversity of light acclimation and photoprotection strategies is anticipated to provide us with valuable material and ideas to help the design of environmentally resilient crop plants.

## Materials and methods

### Plant material and light treatments

Physcomitrella (*P.patens*) Gransden WT strain and the STN7 kinase(s) depleted mosses generated in this work, were grown with a light intensity of 45 µmol photons m^−2^ s^−1^, 24°C, 16/8-h photoperiod as control conditions (CL). Eleven-day-old cultures grown in minimal PpNO_3_ media were used as starting material for short-term light treatments with different light intensity/quality. Two hours low and high white light (2h-LL and 2h-HL) treatments were performed as described previously (Gerotto et al., 2019). For red and FR light treatments, LEDs with emission peaks at 660, 630, or 735 nm were applied for 2 h using Heliospectra L4A light source (Heliospectra AB, Göteborg, Sweden) with a light intensity close to the CL (40–45 µmol photons m^−2^ s^−1^).

For long-term acclimation experiments, 4-d-old plates were moved from CL to either high (long-HL, 600 µmol photons m^−2^ s^−1^), low (long-LL, 7 µmol photons m^−2^ s^−1^) or fluctuating light (long-FL, cycles of 5 min at 25 µmol photons m^−2^ s^−1^/1 min at 800 µmol photons m^−2^ s^−1^ (Gerotto et al., 2016)) with 16/8-h photoperiod as CL and analyzed after 7 d of acclimation to the applied light regime. Samples were collected 6 h after the lights were turned on in growth conditions. In the case of FL, sampling also occurred after 3 min of LL step.

### Generation of stn7 KO in Physcomitrella

The genome of Physcomitrella harbors two genes encoding for kinases homologous to Arabidopsis STN7 kinase (*STN7.1*, Pp3c4_25980; *STN7.2*, Pp3c26_5140). We here generated mosses depleted either in one *STN7* gene (*stn7.1* single KO or *stn7.2* single KO) or both (*stn7* double KO) using targeting constructs that interrupted *STN7.1* and *STN7.2* genes with a resistance cassette, as done previously (Alboresi et al., 2010). Further information on the KO constructs design and the complete list of primers used for the mutants’ generation and screening is reported in Supplemental Table S2. Physcomitrella Gransden WT was used to obtain the single KO lines for *stn7.1* and *stn7.2*. To obtain *stn7* double KO lines, the *STN7.1* gene was knocked-out in *stn7.2* single KO genetic background and *vice versa*, as two independent approaches, to deplete both STN7.1 and STN7.2. Moss transformation was performed as previously (Gerotto et al., 2016). After two rounds of selection, the disruption of *STN7.1* and/or *STN7.2* genes was verified by amplifying the flanking regions of the homologous recombination construct (Left and Right Borders, LB and RB, see Supplemental Table S2) on genomic DNA of resistant lines, extracted with GeneJET Plant Genomic DNA Purification Kit (Thermo Fisher Scientific, Waltham, MA, USA). RNA was purified with RNeasy Plant Mini Kit (Qiagen) and used as a template for cDNA synthesis (RevertAid Reverse Transcriptase, Thermo Scientific) to verify the absence of *STN7.1* and/or *STN7.2* transcripts by RT-PCR in the selected *stn7.1* and/or *stn7.2* KO lines.

### Thylakoid extraction, fractionation, denaturing, and native gel electrophoresis

Thylakoid extracts, SDS-PAGE, immunoblotting, lpBN, and bi-dimensional (2D-lpBN-SDS-PAGE) gel electrophoresis were performed as before (Gerotto et al., 2019).

Thylakoid fractionation was done as in Pinnola et al. (2015) with slight modifications. Briefly, thylakoids at 0.4 mg/mL Chl concentration were incubated with 0.5% digitonin (w/v) for 7 min in constant shaking at 4°C. Unsolubilized material was removed by centrifugation for 5 min at 4,000 g. After diluting the supernatant with an equivalent volume of buffer, the grana plus margins fraction was collected at 40,000 g and the stroma-exposed fraction at 100,000 g.

### Chl fluorescence analyses

Chl *a* fluorescence emission spectra at 77K were monitored from thylakoid suspension diluted to 10 µg Chl/mL in thylakoid storage buffer (50 mM Hepes/KOH pH 7.5, 100 mM sorbitol, 10 mM MgCl_2_, 10 mM NaF) using an Ocean Optics QEPro fiber optic spectrometer. Samples were excited with BL (470-nm filter) to record the emission spectra.

“State transitions” kinetics were recorded in vivo with Dual-PAM-100 spectrofluorometer (Walz) equipped with a Dual-DB and Dual-E measuring heads providing blue measuring and actinic light and FR light, allowing to induce state 1 and state 2.

Light curve analyses were recorded with Dual-PAM-100 analyzing concomitantly Chl Fluorescence and P700 absorption signals. Twenty steps of increasing actinic light (from 8 to 1,960 µmol photons m^−2^ s^−1^, 60 s each) were applied to CL-grown moss tissues of the different genotypes dark acclimated 40 min before measurements. The parameters were calculated by the Dual-PAM software as follows: Fv/Fm = (Fm−Fo)/Fm; Y(II) = (Fm'−F)/Fm'; qL =  (Fm'−F)/(Fm'−Fo') × Fo'/F; NPQ = (Fm−Fm′)/Fm′; Qa rel red = F/Fm; Y(I) = 1−Y(ND)−Y(NA) = (Pm'–P700ox)/Pm.

### MS

Analysis by MS of the spots excised from 2D-lpBN-SDS-PAGE was performed as in Gerotto et al. (2019). The targeted analysis of the phosphorylations of STN7 in the SDS-PAGE was performed using an inclusion list of the theoretical masses of all the tryptic peptides of STN7 as in Trotta et al. (2016). lpBN excised bands were first run for 0.5 cm in a 6% w/v acrylamide, 6 M urea SDS-PAGE as in Trotta et al. (2016), subjected to in-gel tryptic digestion and further processed as described in Gerotto et al. (2019). The MS proteomics data have been deposited to the ProteomeXchange Consortium via the PRIDE (Perez-Riverol et al., 2019) partner repository with the dataset identifier PXD026183 and 10.6019/PXD026183.

### Statistical analyses

Numerical data are presented as average ± SD of at least three independent biological replicates. Statistical evaluation of numerical differences was analyzed with Sigmaplot (version 14.0) software using all-pairwise multiple-comparison procedures one-way ANOVA (the Holm–Sidak method) with the Shapiro–Wilk method for normality testing and the Brown–Forsythe method for equal variance testing. When equal variance test (Brown–Forsythe) failed, Dunn’s test was used to evaluate the significance of the difference.

## Accession numbers

Sequence data used in this article can be found in the Phytozome data libraries (https://phytozome.jgi.doe.gov/pz/portal.html) under accession number: *P. patens STN7.1*, Pp3c4_25980; *STN7.2*, Pp3c26_5140; *STN8*, Pp3c1_7450; *STN-like*, Pp3c10_6850; accession numbers of other sequences used for multiple sequences alignment and MS analyses are detailed in the Supplemental Material Online (Supplemental Figures S1 and S14; Supplemental Table S1). MS database used in this work is the same as in Gerotto et al. (2019) and includes sequences from Phytozome or UniProt data libraries.

## Supplemental data

The following materials are available in the online version of this article.

**Supplemental Figure S1.** Amino acid sequence alignment of Arabidopsis STN7 with Physcomitrella STN7, STN8 and STN-like kinases.

**Supplemental Figure S2.** Preparative SDS-PAGE for the MS detection of STN7.

**Supplemental Figure S3.** Characterization of *stn7.1* single KO and *stn7.2* single KO.

**Supplemental Figure S4.** Complete light curve kinetics of PSII- and PSI-related parameters.

**Supplemental Figure S5.** State transition kinetics.

**Supplemental Figure S6.** Characterization of thylakoid protein phosphorylation upon short-term exposure to different light quality or quantity.

**Supplemental Figure S7.** LHCB6 monomeric antenna accumulation upon short-term light changes.

**Supplemental Figure S8.** 2D-lpBN-SDS-PAGE of WT and *stn7* double KO from overnight dark acclimated and 2h-HL samples.

**Supplemental Figure S9.** lpBN-PAGE from WT long-term acclimated sample.

**Supplemental Figure S10.** The 77K spectra on thylakoids from long-HL acclimated cultures.

**Supplemental Figure S11.** Time course of maximum PSII quantum efficiency during long-term acclimation.

**Supplemental Figure S12.** 2D-lpBN-SDS-PAGE of thylakoids and grana fraction from long-FL illumination.

**Supplemental Figure S13.** Detection of Thr phosphorylations in PSI SCs (PSI-large and PSI-LHCI-LHCII).

**Supplemental Figure S14.** N-terminus of LHCBM isoforms of Physcomitrella.

**Supplemental Table S1.** List of identified proteins and related peptides in SDS-PAGE bands, lpBN-PAGE bands, and 2D-lpBN-SDS-PAGE spots (Excel file).

**Supplemental Table S2.** Primers used for the generation and screening of *stn7.1* and *stn7.2* KO plants.

**Supplemental Table S3.** MS confirmation of *stn7* double KO lines.

**Supplemental Table S4.** Chl content after long-term acclimation.

## Supplementary Material

kiac294_Supplementary_Data

## Notes

C.G., A.T., and E.-M.A. designed the research. C.G., A.T., and A.A.B. performed the experiments. C.G., A.T., T.M., and E.-M.A. analyzed the data. C.G., A.T., and E.-M.A. wrote the manuscript.

The authors responsible for distribution of materials integral to the findings presented in this article in accordance with the policy described in the Instructions for Authors (https://academic.oup.com/plphys/pages/General-Instructions) are: Eva-Mari Aro (evaaro@utu.fi) and Caterina Gerotto (c.gerotto@univpm.it).
