# Supplementary material for: Role of serine/threonine protein kinase STN7 in the formation of two distinct photosystem I supercomplexes in Physcomitrium patens
Source: Plant Physiol. 2022 Jun 23;190(1):698–713. doi: 10.1093/plphys/kiac294 (PMC9434285; doi:10.1093/plphys/kiac294)
Supplement: kiac294_Supplementary_Data [file kiac294_supplementary_data.zip › Gerotto et al_SI_revised 10june22_Final.pdf]

ROLE OF STN7 KINASE IN FORMATION OF TWO DISTINCT PSI SUPERCOMPLEXES IN *PHYSCOMITRIUM PATENS*

Caterina Gerotto, Andrea Trotta, Azfar Ali Bajwa, Tomas Morosinotto, Eva-Mari Aro

SUPPLEMENTAL DATA

Supplemental Figure S1. Amino acid sequence alignment of Arabidopsis STN7 with Physcomitrella STN7, STN8 and STN-like kinases. Protein sequences of Arabidopsis STN7 (AT1G68830.1) and Physcomitrella STN7.1, STN7.2, STN8 and STN-like kinases (Pp3c4_25980V3.1, Pp3c26_5140V3.1, Pp3c1_7450V3.1, Pp3c10_6850V3.5, respectively) were retrieved from Phytozome (https://phytozome.jgi.doe.gov/pz/portal.html), aligned with ClustalW tool in BioEdit and characterized with bioinformatic tools. The start and the end of the kinase domain profile are highlighted in blue, the Ser/Thr protein kinases active site signature in green, as identified by ScanProsite web tool (prosite.expasy.org). Further, the position of phospho-residues identified in Arabidopsis STN7, i.e. Ser-526, Thr-537 and Thr-541 (Trotta et al., 2016) and in Physcomitrella STN7 (this work, Thr-569 and Thr-573 in STN7.1) are highlighted with a yellow/red labelled arrow and are all located in the C-terminus of the sequences (see positions 653, 663 and 667 of the alignment). In addition to the well-known STN7 and STN8 kinases, in Physcomitrella a fourth gene, named STN-like (Pp3c10_6850), was retrieved with blast tool at Phytozome (phytozome.jgi.doe.gov) showing similarity with STN7 (43-45%) and STN8 (39%). The gene of the STN-like protein (Pp3c10_6850) is predicted in seven transcript isoforms (V3.1-3.7), only one of those is shown here. The chloroplastic transit peptide of STN-like protein as predicted by ChloroP software (http://www.cbs.dtu.dk/services/ChloroP/) is shown as underlined.

```
                                       10        20        30        40        50        60        70
                              ....|....|....|....|....|....|....|....|....|....|....|....|....|....|
At STN7_AT1G68830.1           ----MATISPGG--------------AYIGTPSPFLGKKLK---------------PFSLTSPILSFKPT
Pp_STN7.1_Pp3c4_25980V3.1     MDTAMACTSGRA---FSLLDVAAVRLGSLPTRRSCAEQKLVVKWKG---EVLGLGPGLRLGLR-LGVSTR
Pp_STN7.2_Pp3c26_5140V3.1     --MAMACRSSRSPSWYEFSDVAVARVPQLTSRGRITGQKLVGQWP------RGLGLGLRVVGRGLSVRRG
Pp_STN8_Pp3c1_7450V3.1        --MGLQLNPSRATVIIGVNAVNVPSVSASGLQLNRTGGCLSSGCRSGFLGCEGLKAAVQLCPAHFGSAKG
Pp_STN like_Pp3c10_6850V3.5   ----MTVMAASLS---------FPSPSTVSLRSSLFRPCLIRRDSQ---------LTYRILPSRLGSIDP

                                       80        90       100       110       120       130       140
                              ....|....|....|....|....|....|....|....|....|....|....|....|....|....|
At STN7_AT1G68830.1           -------VKLN--SSCRA--QLIDTVHNLFIGVGVGLPCTVMECGDMIYR-----------STLPKSNGL
Pp_STN7.1_Pp3c4_25980V3.1     -------GRRGKKAVCRASFELVEHLHGLVLGVGVGLPCTVMECGDVVYR-----------STLPR-QGF
Pp_STN7.2_Pp3c26_5140V3.1     -------TSRRAIVRVRASFELAEHLHGLVVGVGVGLPCTVMECGDVVYR-----------STLPR-QGF
Pp_STN8_Pp3c1_7450V3.1        KGNGHGKVRVAVDVVATAVTDVIMQQLPFIDSLQHGMPELYSNLPDVASGGLSELANAGAVKSLVELSDF
Pp_STN like_Pp3c10_6850V3.5   -------------GRCVAQISVAGFSEALQSGLGPEAYQSMTKLYNDVFVN-------YAKLGVHLPDDE
```

```
                                                                                 Start kinase domain
                                       150       160       170       180       190       200       210
                               ....|....|....|....|....|....|....|....|....|....|....|....|....|....|
At STN7_AT1G68830.1            TITAPGVALALTALSYLWATPGVAPGFFDMFVLAFVERLFR-----PTFRKDDFVVGKKLGEGSFGVVYK
Pp_STN7.1_Pp3c4_25980V3.1      QITTPGVALTVLIVTYLWATPGVAPGFWDMFVLAPLENLFR-----PMFKKEDLNLGKKLGEGAFGTVYK
Pp_STN7.2_Pp3c26_5140V3.1      QITTPGVALTVLIVTYLWATPGVAPGFWDMFVLAPLENLFR-----PVFKKEDLSLGKKLGEGAFGTVFK
Pp_STN8_Pp3c1_7450V3.1         ERYRAAGALGIG-FIYLTAKPGVLKGAFDMYIGAPAQAAIENLRGRRSWKRTDFVIDQRLGEGSFGTVYT
Pp_STN like_Pp3c10_6850V3.5    NAVGYLAVALPAALLYLTATPGPIAGLLDFVRDRADVNRSL------TFRAHEVKIGRLMGEGSFGIAYE

                                       220       230       240       250       260       270       280
                               ....|....|....|....|....|....|....|....|....|....|....|....|....|....|
At STN7_AT1G68830.1            VSLS-KKRSNEE-------------------------GEYVLKKATEYGAVEIWMNERVRRACGNSCADF
Pp_STN7.1_Pp3c4_25980V3.1      ASLVNKQLLKKD-------------------------GPLVVKKANEYGAVEAWMNERVRRACRKSCADF
Pp_STN7.2_Pp3c26_5140V3.1      ASLSNKQLLKKD-------------------------GPLVVKRANEYGAVEAWMNERVRRACRKSCADF
Pp_STN8_Pp3c1_7450V3.1         GVILPKGVNPDEEFGRRGRRLEEFEDYKKFKRVILKKVKVGVVGAEECGEMEEWFNYRMTRAAPDVCAKF
Pp_STN like_Pp3c10_6850V3.5    GFIHKGGKRPGKQP----------------LHVVLKKNKARVAGANQMLNAEIHMNQRLQRTSPEAIADF

                                       290       300       310       320       330       340       350
                               ....|....|....|....|....|....|....|....|....|....|....|....|....|....|
At STN7_AT1G68830.1            VYGFLDKSSK---KGP-EYWLLWKYEGESTLAGLMQSKEFPYNVETIILG--KVQDLPKGLE--RENKII
Pp_STN7.1_Pp3c4_25980V3.1      IHGFLDETSS---NGKDEFWLLWRYEGSSTLADLMGSRDFPYNVEELLLGPGKGGDLPRGPE--RQNRII
Pp_STN7.2_Pp3c26_5140V3.1      VHGFLHETSS---KGKEEFWLLWRYEGNATLADLMANRDFPYNVEELILGPRKGEDLPRGPE--RQNRIV
Pp_STN8_Pp3c1_7450V3.1         LGTFTADITKGQ-FTAGGKWLIWMYEGDSTLLDFMKQQNFPQNLEVPLFG--RTLNNDDEIK--RNSLII
Pp_STN like_Pp3c10_6850V3.5    LGTVNVSSSQARGKLTEGVWLVWKYQGHWSLDHYMKQKNFPENIAEAVLGQKVKNTASRAALNKQNALVV

                                           Active site sign.
                                       360       370       380       390       400       410       420
                               ....|....|....|....|....|....|....|....|....|....|....|....|....|....|
At STN7_AT1G68830.1            QTIMRQLLFALDGLHSTGIIHRDVKPQNIIFSEGSRSFKIIDLGAAADLRVGINYIPKEFLLDPRYAAPE
Pp_STN7.1_Pp3c4_25980V3.1      RITMRQILSALAQLHSTGIVHRDIKPQNIIYSEETKSFKIIDLGAAADLRVGINYIPKEFLLDPRYSAPE
Pp_STN7.2_Pp3c26_5140V3.1      RSIMRQILSALAQLHATGIVHRDIKPQNIIFSEETKSFKIIDLGAAADLRVGINYIPKEFLLDPRYSAPE
Pp_STN8_Pp3c1_7450V3.1         TQIMRQIITCLKKMHAVGIIHRDVKPSNVVVTDKGK-LKFIDFGAATDLRVGKNYVPDRGILDPDYCPPE
Pp_STN like_Pp3c10_6850V3.5    RKIMQQILTNLRDLHRSGVVHRDLKPLNLVLSEDSGFFKLIDLGACVDIRSGFNYDPFETVIDPTYAAPE

                                       430       440       450       460       470       480       490
                               ....|....|....|....|....|....|....|....|....|....|....|....|....|....|
At STN7_AT1G68830.1            QYIMSTQTPSAPSAPVAAALSPVLWQMNLPDRFDIYSIGLIFLQMAFPSLRSDSNLIQFNRQLKRCDYDL
Pp_STN7.1_Pp3c4_25980V3.1      QYIMSTQTPSAPPAIIAAALSPVLWQMNLPDRFDIYSLGLIYLQMAFPNLRSDSGLISFNRQLKRRDYDM
Pp_STN7.2_Pp3c26_5140V3.1      QYIMSTQTPSAPPAIIAAALSPVLWQMNLPDRFDIYSAGLIYLQMAFPNLRTDSGLISFNRQFKRRDYDL
Pp_STN8_Pp3c1_7450V3.1         LYVLPEETPLPPPAPVAAILSPLLWQLNSPDLFDMYSIGVIFLQMCCVGLRTSIGLQTFKKEIESVGYNL
Pp_STN like_Pp3c10_6850V3.5    HYVMPTSTPTLPPDPLCSMISPLVWFLNTPDRFDLYSAGLILMQLCVKQLRQDVGLKTFSTQFKRDGYDL

                                                                                 End Kinase domain
                                       500       510       520       530       540       550       560
                               ....|....|....|....|....|....|....|....|....|....|....|....|....|....|
At STN7_AT1G68830.1            TAWRKLVEPRASADLRRGFELVDLDGGIGWELLTSMVRYK-----------ARQRISAKAALAHPYFD--
Pp_STN7.1_Pp3c4_25980V3.1      VEWRKLVEAKQSQDIKQGFEILDMDSGVGWELVQDMLRFK-----------GRKRISANAALAHPYFS--
Pp_STN7.2_Pp3c26_5140V3.1      VKWRSLVEGKPNQDIMQGFEILDLDDGVGWELVQDMLRFK-----------GRKRISANAALAHPYFE--
Pp_STN8_Pp3c1_7450V3.1         QKWRDITKVARIN-----FDLLDADGGKGWDLATKLVCGRN--------AFNRGRLSAESALRHPYFL--
Pp_STN like_Pp3c10_6850V3.5    DVWRKRCSISSEE-----FAVLDADDGAGWELAKAMLQPRHDKAFFIWPSLRSSRPSAAAALRHRFIRGT

                                       570       580       590       600       610       620       630
                               ....|....|....|....|....|....|....|....|....|....|....|....|....|....|
At STN7_AT1G68830.1            --------------------RQGLLALSVMQNLRMQYFRATQQDYSEAANWVIQLMAKNGTEKDGGFTET
Pp_STN7.1_Pp3c4_25980V3.1      --------------------REGLLGLSIMQRLRMNVLRGAQPD-NEGVDWVTSLMAKSGTDSVGGFTEA
Pp_STN7.2_Pp3c26_5140V3.1      --------------------RAGFLGLSVMQRLRLNLLRGSQQD-NQGVYWVTSLMAKSGTDSVGGFTEA
Pp_STN8_Pp3c1_7450V3.1         --------------------LGGDQASTIISKVTFSK---------------------------------
Pp_STN like_Pp3c10_6850V3.5    FLPRRINIASLAPDITLLPSLPSLPSLPSLSQLLRPVFSGESAKEAAVKQLTNLREASGNINPKRVIIALGAA

                                       640       650 P     660 P  P670       680       690       700
                               ....|....|....|....|..↓.|....|...↓|..↓.|....|....|....|....|....|....|
At STN7_AT1G68830.1            QLQELREKEPRKKANAQRNALASALRLQRKLVKTVTETI---------------DEISDG---RKTVWWN
Pp_STN7.1_Pp3c4_25980V3.1      QLQSMREKEPKVKGSVRRNAMAQALRIQRKAARTIAATV---------------DEIQKGEGAKRVNWWN
Pp_STN7.2_Pp3c26_5140V3.1      QLQNIREKEPVTKGSVRRNAMAQALRLQRKAARTIAATV---------------DEIQKGDVVKRVNWWN
Pp_STN8_Pp3c1_7450V3.1         ----------------------------------------------------------------------
Pp_STN like_Pp3c10_6850V3.5    LPTAATSAFVLTIGWVTLATLKSSTHISYELGRSVANSVGLSGSACLVFFLFINVQSDEAAFHGPTAWSI

                                       710
                               ....|....|....|
At STN7_AT1G68830.1            RWIPREE--------
Pp_STN7.1_Pp3c4_25980V3.1      RWQN-----------
Pp_STN7.2_Pp3c26_5140V3.1      RWQN-----------
Pp_STN8_Pp3c1_7450V3.1         ---------------
Pp_STN like_Pp3c10_6850V3.5    TFQDRAFRSCVSHGG
```

Supplemental Figure S2. Preparative SDS-PAGE for the MS detection of STN7. Thylakoid proteins were isolated from Physcomitrella WT, *stn7* double KO lines 1 and 2 (*stn7* dKO#1 and *stn7* dKO#2 , respectively) cultures dark-acclimated overnight, separated in 12% acrylamide 6 M urea SDS-PAGE and stained with coomassie. A lane with thylakoid proteins from Arabidopsis was run in parallel as a reference. The band corresponding to MW of 48 kDa contains STN7 and STN8 (Trotta et al., 2016; Gerotto et al., 2019). Such band was excised from the different samples, subjected to in-gel trypsin digestion and analyzed by mass-spectrometry based proteomics. This analyses confirmed the lack of STN7 protein in the two *stn7* double KO lines, while in WT the protein was found phosphorylated (Threonines at positions 663 and 667 of the alignment in Supplemental Figure S1).

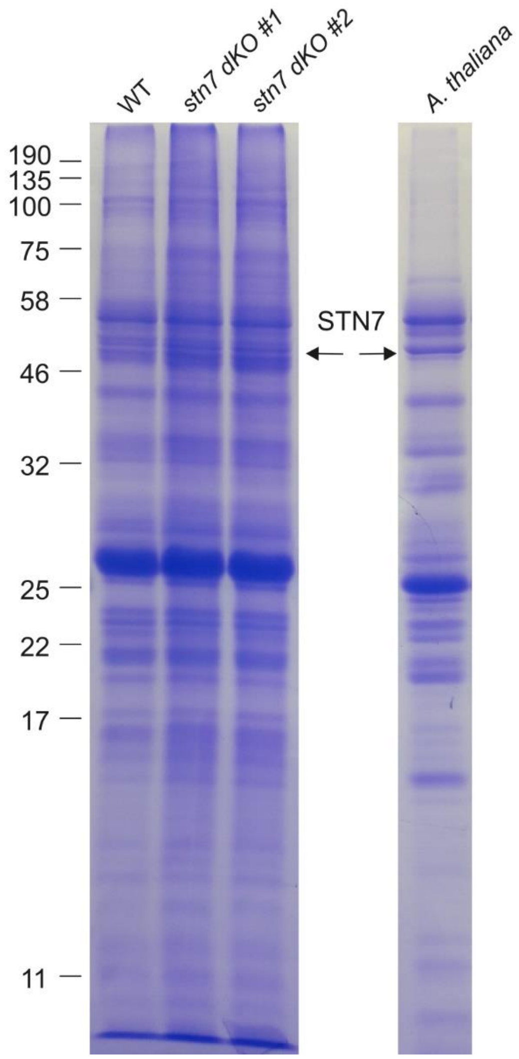

Supplemental Figure S3. Characterization of *stn7.1* single KO and *stn7.2* single KO. The characterization of two independent clones each for *stn7.1* single KO (*stn7.1* sKO) and *stn7.2* single KO (*stn7.2* sKO) genotypes is shown. Lines *stn7.1* sKO #1 and *stn7.2* sKO #1 are the ones then used a genetic background for the generation of *stn7* double KO, the characterization of which is shown in Figure 1. **A)** Verification of *STN7.1* and *STN7.2* accumulation at transcript level in *stn7.1* single KO and *stn7.2* single KO genotypes, showing in each only one of the isoforms is not expressed, as expected in the single mutants. Details on the primers used are provided in Supplemental Table S2. **B)** Anti-P-Thr immunodetection on thylakoid extracts. Plants were grown in CL for 11 days, dark-acclimated overnight (D) and exposed to 2h of low light intensity (2h-LL), a condition that maximally induces LHCBM phosphorylation in the WT (see Supplemental Figure S6). 1 µg of Chl was loaded in each lane. **C)** lpBN-PAGE profile after solubilization of the same samples as (B). Thylakoids were solubilized with 1% β-DM at a final Chl concentration of 0.5 µg/µl. A total of 6 µg of Chl was loaded in each lane. The main photosynthetic complexes, as identified in previous work (Gerotto et al., 2019), are indicated. The PSI supercomplexes SC4 (PSI-large) and SC6 (PSI-LHCI-LHCII) are highlighted with red labels. In (B) and (C) the single *stn7.1* single KO and *stn7.2* single KO are indistinguishable from the WT.

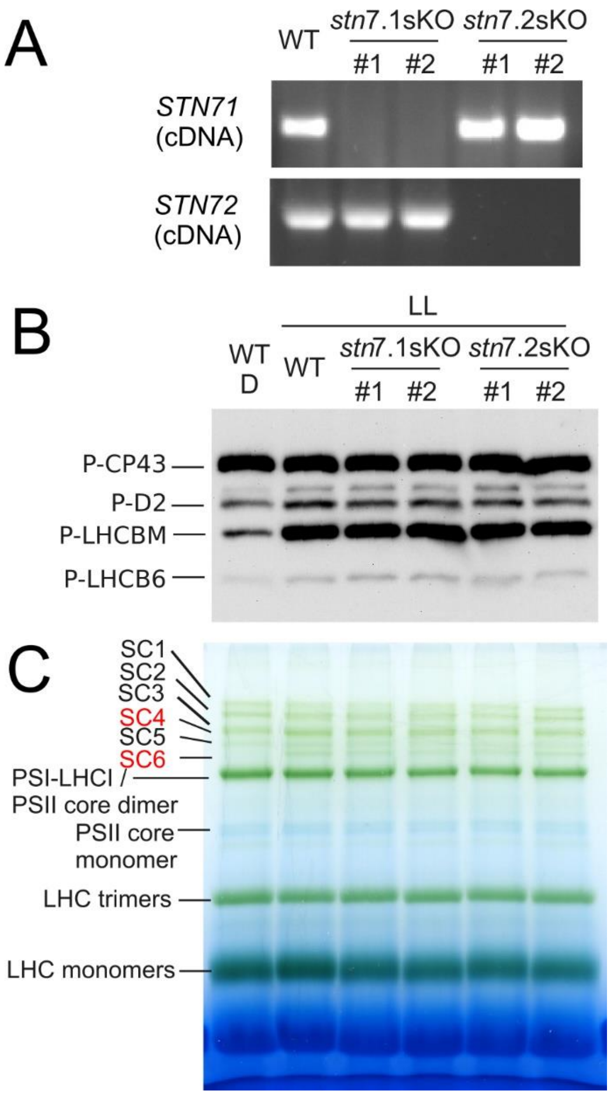

Supplemental Figure S4. Complete light curve kinetics of PSII- and PSI-related parameters. A) Fv/Fm of dark acclimated (40 min) CL-grown tissue of WT (n=23) and *stn7* double KO lines (n=17 each). Both *stn7* double KO lines Fv/Fm are statistically different from the WT (ANOVA, p<0.01). **B-F)** Complete light curve kinetics of Y(II) in (B), Y(I) in (C), Q_A relative reduction in (D), qL in (E) and NPQ in (F). For (D-F), the data in the range 0-500 µmol photons m^{-2} s^{-1} are the ones shown also in Figure 1C-E. Parameters from Light curve kinetics are shown as average ± SD for WT (black, n=6), *stn7* double KO line 1 (red; n= 5) and *stn7* double KO line 2 (orange, n=5). Statistical evaluation for PSII related parameters is shown in Figure 1. In the case of Y(I), values were not statistically different between WT and *stn7* double KO lines at any light intensity. Data are shown as average ± SD.

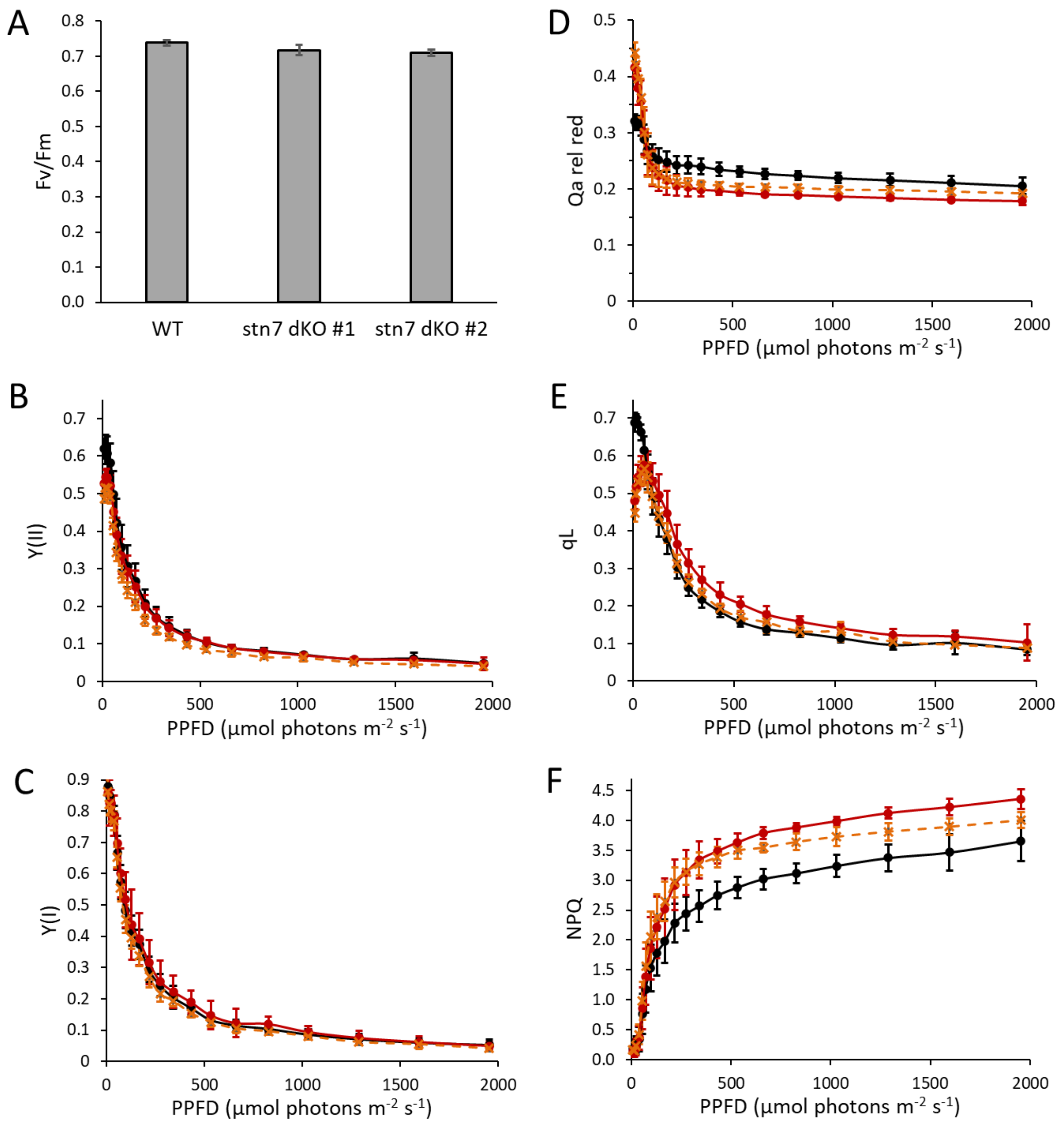

Supplemental Figure S5. State transition kinetics. Representative fluorescence chart of dark acclimated WT (A), *stn7* double KO line 1 (B) and line 2 (C) exposed to low intensity blue light (BL), followed by far red (FR) illumination (inducing state 1, Fm1), and again BL (inducing state 2, Fm2) as done with other plant species (Bellafiore et al., 2005; Betterle et al., 2015). BL illumination was kept during all measurement as indicated with the blue bar on the x axis, FR illumination is shown as the red bar. In the different samples, fluorescence charts were normalized to the initial Fm value. Differences in the state transitions kinetics resemble the results in other plant species (Bellafiore et al., 2005; Betterle et al., 2015), with a reduced fluorescence quenching in the BL phase in the *stn7* double KO with respect to the WT. Also, Fm' at the end of FR and second BL phases (Fm1 and Fm2, respectively) were different in the WT but not in the *stn7* double KO lines.

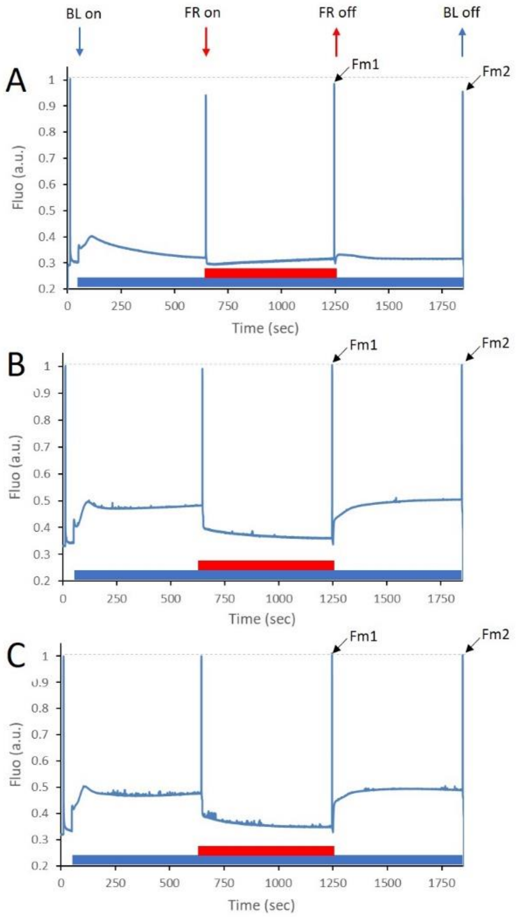

Supplemental Figure S6. Characterization of thylakoid protein phosphorylation upon short-term exposure to different light quality or quantity. Starting samples for all light treatments were 11-days old WT mosses, grown in CL conditions and dark-acclimated overnight prior to light treatment. **A)** “state 2” induction: LL indicates mosses treated with white light of low intensity, 660 and 630 the wavelength of the red light used, 660 nm and 630 nm, respectively. All treatments lasted 2 h starting from dark acclimated mosses (D). LL induced the maximal LHCBM phosphorylation. Illumination with 630 nm had a more pronounced effect on LHCBM phosphorylation than exposure to 660 nm. **B)** “State 1” induction: FR: far red light (735 nm), 10 min treatments of dark acclimated mosses (D). **C)** Dynamics of phosphorylation upon white light changes. Dark acclimated mosses (D) were exposed to 2 h LL (LL1, 7 µmol photons m^{-2} s^{-1}), then to 2 h HL (HL, 500 µmol photons m^{-2} s^{-1}), followed by other 2 h of LL (LL2, 7 µmol photons m^{-2} s^{-1}). 1 µg of Chl was loaded in each lane. **D)** 77k fluorescence spectra of the thylakoids from WT moss treated as in (A) and (B).

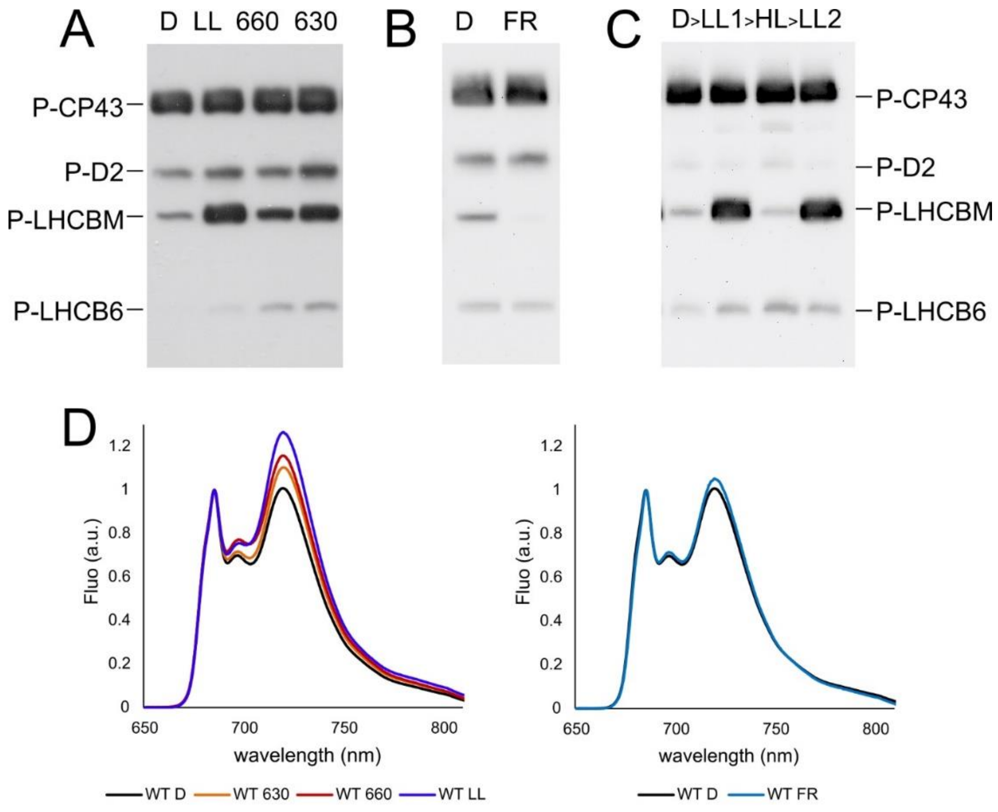

Supplemental Figure S7. LHCB6 monomeric antenna accumulation upon short-term light changes. Immunoblotting with anti-LHCB6 antibody (Agrisera) on thylakoid extracts of WT and the two independent *stn7* double KO lines (*stn7* dKO #1 and #2). Samples grown in CL were dark acclimated overnight (D), then exposed for 2 h to LL (LL, 7 µmol photons m^{-2} s^{-1}) and subsequently for 2 h to HL (HL, 500 µmol photons m^{-2} s^{-1}) prior to thylakoid extraction. 1 µg of Chl was loaded in each lane. Coomassie staining of the PVDF membrane is also shown as a loading control. The picture shows an equal amount of LHCB6 protein irrespective of the light treatment and genotype, confirming the different P-LHCB6 signals obtained by cross-reaction with anti-P-Thr antibody shown in Figure 2A are due to a different phosphorylation level of LHCB6 protein, not to an altered LHCB6 protein expression.

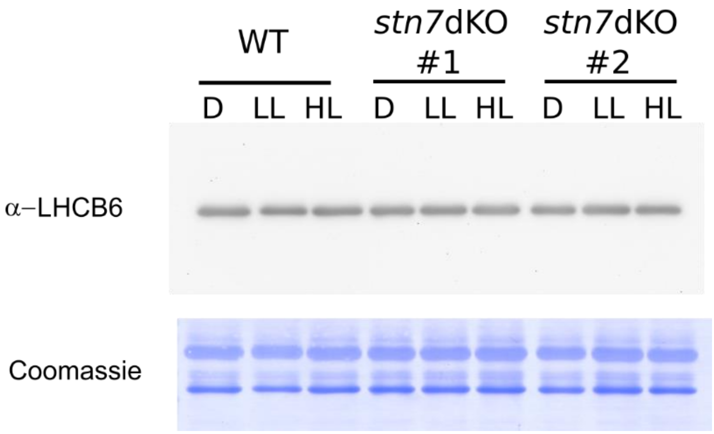

Supplemental Figure S8. 2D-lpBN-SDS-PAGE of WT and *stn7* double KO from overnight dark-acclimated and 2h-HL samples. Additional 2D-lpBN-SDS-PAGE data on short-term white light treatment. Thylakoids were extracted from moss tissues after overnight dark acclimation (this figure, gel panels on top), after the following 2h-LL exposure (results shown in Figure 3B-C) and after the subsequent 2h-HL treatment (500 µmol photons m^{-2} s^{-1}, this figure, bottom gels). Thylakoids were first solubilized with 1% β-DM at a final Chl concentration of 0.5 µg/µl. A total of 6 µg of Chl was loaded in each lpBN lane used for the 2D electrophoresis shown here. WT and *stn7* double KO line 2 are compared. Protein spots were stained with Sypro Ruby (gels on the left) while phospho-proteins were detected with ProQ Diamond (gels on the right). The major protein subunits of the thylakoid protein complexes are indicated as previously identified (Gerotto et al., 2019). The red asterisks indicated the position of the PSI-Large complex (SC4).

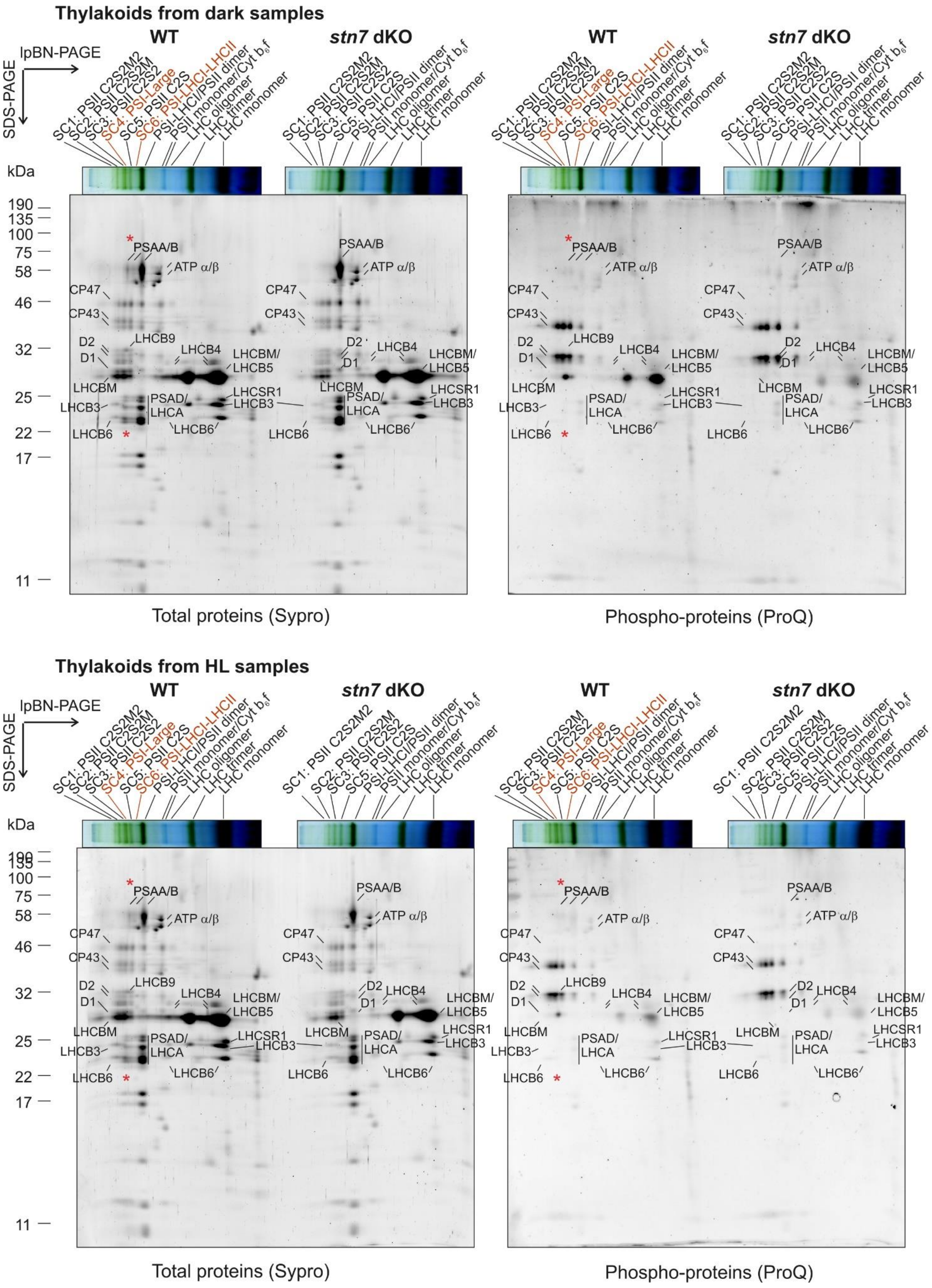
Thylakoids from dark samples
WT
stn7 dKO
WT
stn7 dKO
lpBN-PAGE
SDS-PAGE
SC1: PSII C2S2M2
SC2: PSII C2S2M
SC3: PSII C2S2
SC4: PSI-Large
SC5: PSII C2S
SC6: PSI-LHCI-LHCII
PSI-LHCI/PSII dimer
PSII monomer/Cyt b6f
LHC oligomer
LHC trimer
LHC monomer
kDa
190
135
100
75
58
46
32
25
22
17
11
PSAA/B
ATP α/β
CP47
CP43
D2
D1
LHCB9
LHCB4
LHCBM/
LHCB5
LHCBM
LHCSR1
LHCB3
PSAD/
LHCA
LHCB6
Total proteins (Sypro)
Phospho-proteins (ProQ)
Thylakoids from HL samples
WT
stn7 dKO
WT
stn7 dKO
Total proteins (Sypro)
Phospho-proteins (ProQ)

Supplemental Figure S9. lpBN-PAGE from WT long-term acclimated samples. Thylakoids were extracted from WT mosses grown in control conditions (CL) or acclimated for 7 days to the following light regimes: LL (long-LL, 7 µmol photons m^{-2} s^{-1}), HL (long-HL, 600 µmol photons m^{-2} s^{-1}) or FL light (long-FL, cycles of 5 min at 25 µmol photons m^{-2} s^{-1}/1 min at 800 µmol photons m^{-2} s^{-1}, as in Gerotto et al., 2016) with the same 16/8 h photoperiod as CL. Thylakoids were solubilized with 1% β-DM at a final Chl concentration of 0.5 µg/µl and a total of 6 µg of Chl was loaded in each lane. The main photosynthetic complexes, as identified in previous work (Gerotto et al., 2019), are indicated. Red labels highlight PSI supercomplexes bands. Separate panels indicate samples were loaded on different lpBN gels, each of them including the CL sample as a reference. The dashed line between CL and FL samples instead indicates those two samples were separated by other lanes in the original lpBN-PAGE gel.

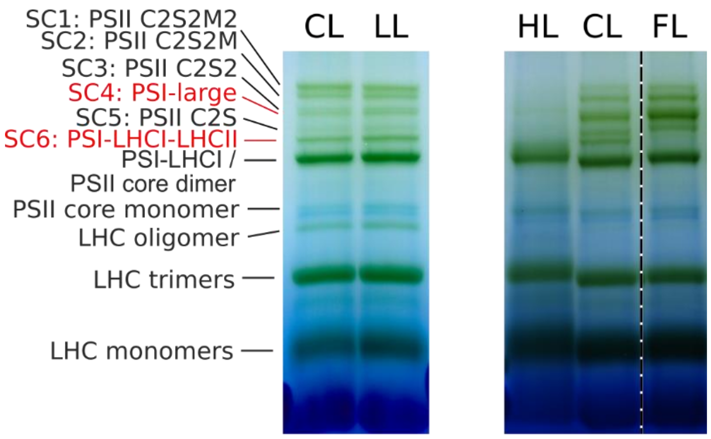

Supplemental Figure S10. 77K spectra on thylakoids from long-HL acclimated cultures. WT, *stn7* double KO line 1 and 2 acclimated for 7 days to HL (HL long; 600 µmol photons m^{-2} s^{-1}) are shown black, red and orange, respectively. The spectra are normalized to the emission peak at 685 nm and are shown as an average of 4 experiments (with thylakoids extracted from 2 independent biological replicates); SD is also shown for the peaks of emission. WT CL from Figure 1 is also shown as a comparison in dashed grey line.

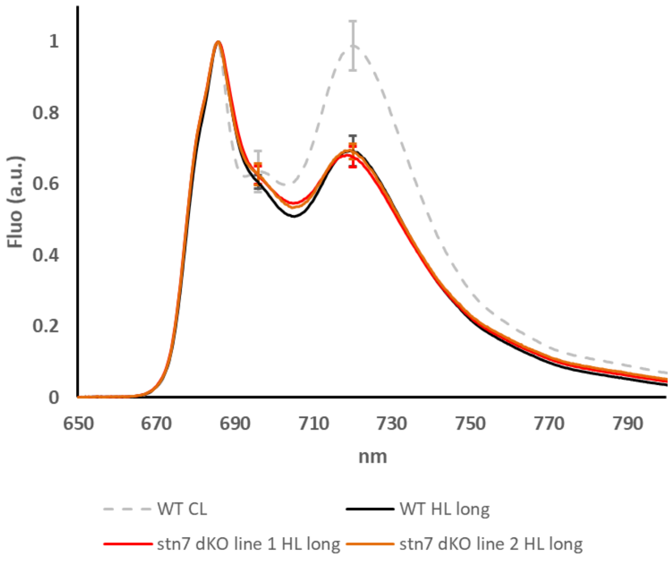

Supplemental Figure S11. Time course of maximum PSII quantum efficiency during long-term acclimation. For each light regime, the figure shows the time course of PSII quantum yield (Fv/Fm parameter) during long-term acclimation of the WT and *stn7* double KO lines #1 and #2. Fv/Fm values were recorded with Imaging PAM (FluorCam, PSI) prior to the moving of 4-days-old plates to the respective light regime (T=0), and after 1, 3 and 7 days of acclimation to LL (7 µmol photons m^{-2} s^{-1}, top right panel), FL (cycles of 5 min at 25 µmol photons m^{-2} s^{-1}/1 min at 800 µmol photons m^{-2} s^{-1}, bottom left panel) or HL (600 µmol photons m^{-2} s^{-1}, bottom right panel). Plates kept in control growth conditions (CL, 45 µmol photons m^{-2} s^{-1}, top left panel) are also shown as a reference. Data are shown as average ± SD of 4 to 13 independent replicates. Two asterisks indicate both *stn7* double KO lines statistically differ from the WT, one asterisk that only one line differs from the WT (ANOVA, Blue asterisks, $p<0.05$; black asterisks $p<0.01$; green asterisks $p<0.001$); except for the sample indicated as # (see day 1, HL), the values from the two *stn7* double KO lines are not statistically different. CL values (T=7 days) are slightly different from the ones reported in Supplemental Figure S4A as the latter were obtained with Dual-PAM-100 device (Walz), while the values presented here were obtained with Imaging PAM (FluorCam, PSI). Notably, the differences in Fv/Fm between WT and the two *stn7* double KO lines grown in CL are present independently of the device employed.

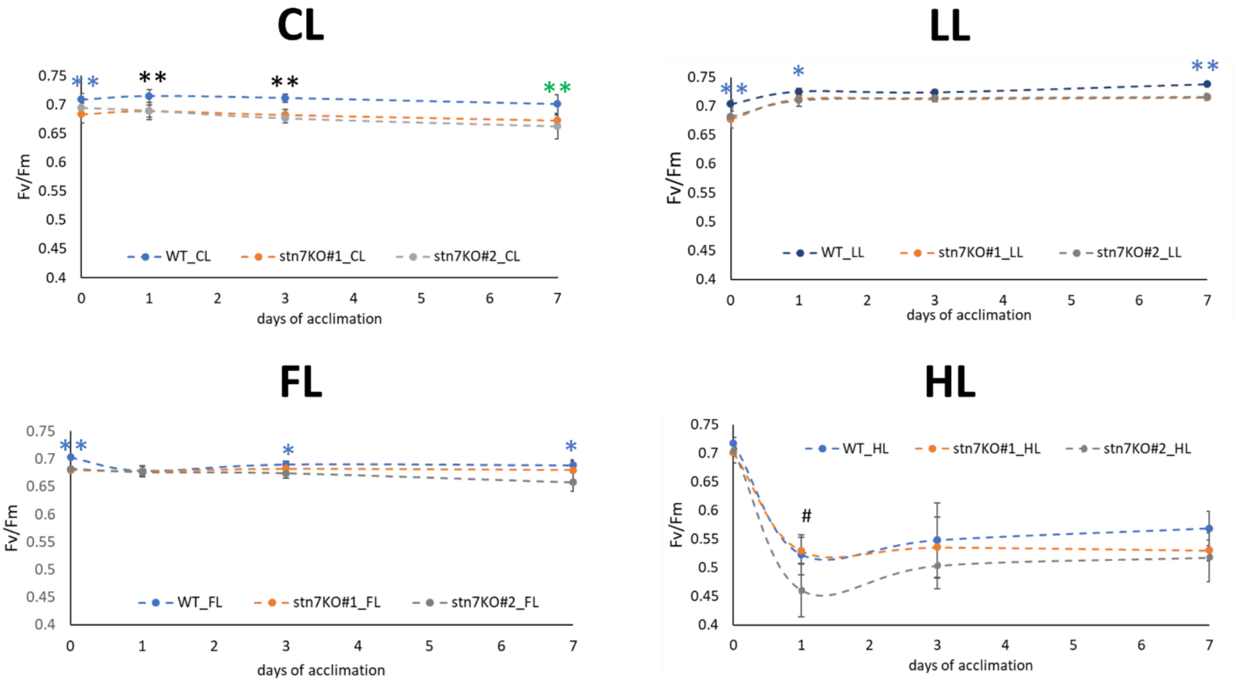

Supplemental Figure S12. 2D-lpBN-SDS-PAGE of thylakoids and grana fraction from long-FL illumination. Analysis by 2D-lpBN-SDS-PAGE of the thylakoid proteins isolated from WT and *stn7* double KO after long-FL (cycles of 5 min at 25 µmol photons m^{-2} s^{-1}/1 min at 800 µmol photons m^{-2} s^{-1}, upper panels), and of the grana + margins fraction (40.000 g pellet) obtained after solubilization with digitonin of the same samples (lower panels). Stroma lamellae fraction 2D-lpBN-SDS-PAGE is instead shown in Figure 6. Upper panels: the pattern and intensity of protein subunits in intact thylakoids from long-FL was very similar to those shown for the short-term 2h-LL thylakoids presented in Figure 3, except for a negligible amount of the PSI-LHCI-LHCII (state transition complex) in long-FL WT samples (SC6), as noted already from the lpBN-PAGE (Figure 5, Supplemental Figure S9). Lower panels: 2D-lpBN-SDS-PAGE from grana fraction showed PSII SC enrichment together with a low amount of PSI and a very faint signal from ATP synthase subunits. No major differences were detected among WT and *stn7* double KO upon Sypro staining, whereas faint LHCBM phosphorylation was again visible with ProQ in *stn7* double KO.

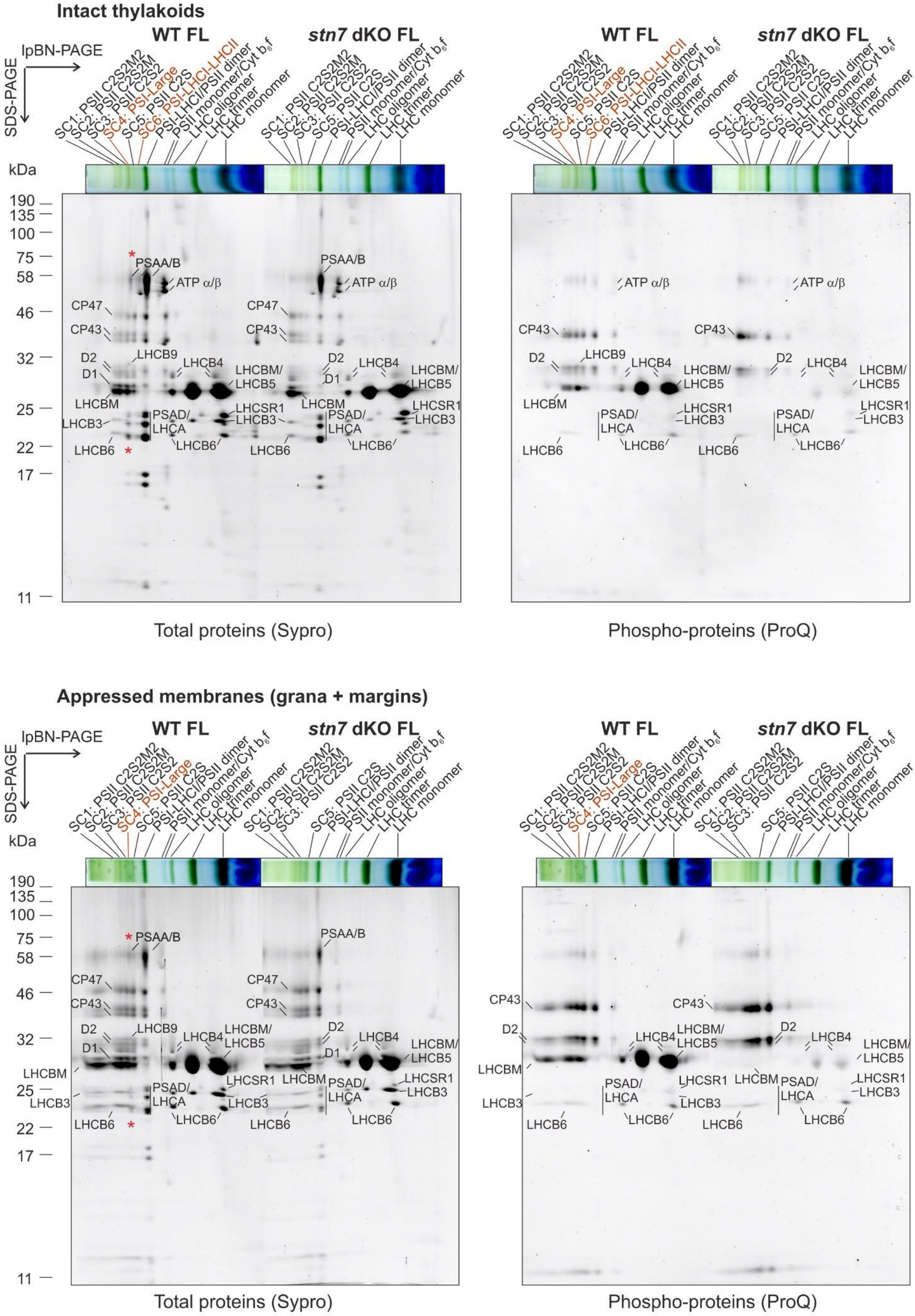
Intact thylakoids
WT FL
stn7 dKO FL
lpBN-PAGE
SDS-PAGE
SC1: PSII C2S2M2
SC2: PSII C2S2M
SC3: PSII C2S2
SC4: PSI-Large
SC5: PSII C2S
SC6: PSI-LHCI-LHCII
PSI-LHCI/PSII dimer
PSII monomer/Cyt b6f
LHC oligomer
LHC trimer
LHC monomer
kDa
190
135
100
75
58
46
32
25
22
17
11
PSAA/B
ATP α/β
CP47
CP43
D2
D1
LHCB9
LHCB4
LHCBM/
LHCB5
LHCBM
LHCSR1
LHCB3
PSAD/
LHCA
LHCB6
Total proteins (Sypro)
Phospho-proteins (ProQ)
Appressed membranes (grana + margins)

Supplemental Figure S13. Detection of Thr phosphorylations in PSI supercomplexes (PSI-large and PSI-LHCI-LHCII). lpBN gel bands of PSI-large (SC4) or PSI-LHCI-LHCII (SC6) were cut, solubilized with Laemmli buffer, loaded onto SDS-PAGE and immunodetected with anti-P-Thr antibody. SC4 band was isolated from stroma lamellae fraction (S), to avoid contamination with nearby PSII SC bands (see Figure 5). All the other bands were instead obtained from lpBN-PAGE from thylakoid samples (Thyl), including the PSII supercomplex $C_2S_2M_2$ (SC1) which was added as a reference for PSII supercomplexes. The sample labelled as "SC6* - *stn7* KO" indicates the gel region corresponding to the WT SC6 (PSI-LHCI-LHCII) was excised from *stn7* double KO lpBN lane and analyzed. Thylakoids obtained from CL (45 µmol photons m^{-2} s^{-1}) or long-LL (LL, 7 µmol photons m^{-2} s^{-1}) treated mosses were used in this experiment.

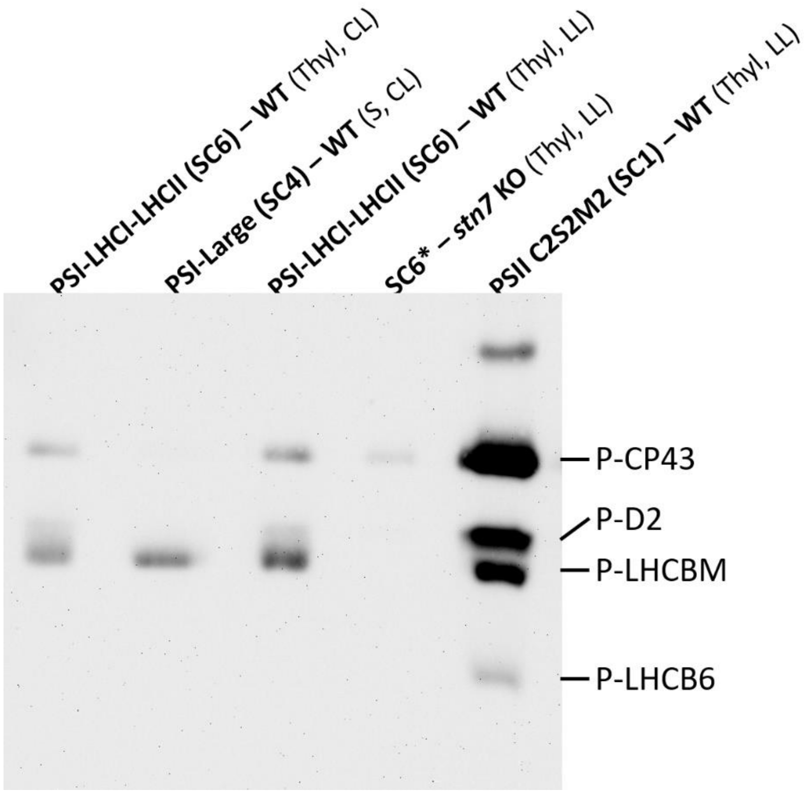

Supplemental Figure S14. N-terminus of LHCBM isoforms of Physcomitrella. Sequence alignment of Physcomitrella LHCBM isoforms with Arabidopsis LHCB1 and LHCB2 and Chlamydomonas LHCBM isoforms (LHCBM5 was excluded from the figure because its N-terminal sequence creates an 8 residues gap after the position 20 of the alignment). The figure shows only the part of the sequence including the N-terminus of the mature proteins (residues interval used is indicated in brackets) and their (putative) phospho-sites. As a reference, the region of the N-terminus which is also shown in Figure 7 is highlighted in bold, the peptides detected by MS in this work are underlined, and all the detected Physcomitrella LHCBM phospho-sites are highlighted in yellow, irrespective from the sample on which they were found. N-terminal positively charged amino acids required for STN7 recognition (RR or RK) are shown in blue letters. For an easier comparison of the position of Thr and Ser residues in the different sequences/species, all Thr and Ser in the sequences are evidenced with red color, with no reference to their phosphorylation status (see Grieco et al., 2016 for a list of detected phospho-sites in Arabidopsis and Chlamydomonas photosynthetic subunits).

```
                                       10        20        30        40        50        60
                               ....|....|....|....|....|....|....|....|....|....|....|....|
Lhcbm1_Pp3c18_8100V3.1         NV--EARISMRKTASKS---SDSIWYGADRPKYLGPFS-GETPSYLNGEFAGDYGWDTAG (30-83)
Lhcbm2_Pp3c21_3950V3.1         SS--EARVTMRRTVKST---SDSIWYGADRPKYLGPFS-GETPSYLTGEFAGDYGWDTAG (30-83)
Lhcbm3_Pp3c6_12510V3.1         AG--EARVTMRKTVSKSSG-SDSIWYGADRPKFLGPFS-GETPSYLNGEFAGDYGWDTAG (31-86)
Lhcbm4_Pp3c13_5930V3.1         SS--QARVTMRRTVNKSAG-SDSIWYGADRPKYLGPFS-GETPSYLNGEFAGDYGWDTAG (30-85)
Lhcbm5_Pp3c13_7900V3.1         NV--QARVTMR----KASS-SDSIWYGADRPKYLGPLS-GETPSYLTGEFAGDYGWDTAG (30-81)
Lhcbm6_Pp3c26_6740V3.1         TS--EARVSMR----KSSG-SDSIWYGADRPKFLGPFS-GETPSYLNGEFAGDYGWDTAG (30-81)
Lhcbm7_Pp3c5_7180V3.1          AN--EARVTMR----KSAG-SDSIWYGADRPKFLGPFS-GETPSYLNGEFAGDYGWDTAG (30-81)
Lhcbm8_Pp3c3_4100V3.1          NV--DSRVTMRRTVNKSAG-SDSIWYGADRPKFLGPFS-GETPSYLNGEFAGDYGWDTAG (30-85)
Lhcbm9_Pp3c2_35930V3.1         SS--QARVSMRKTVSKSSG-SDSIWYGADRPKWLGPFS-GETPSYLNGEFAGDYGWDTAG (29-84)
Lhcbm10_Pp3c22_5610V3.1        TS--QARVTMRKTVSKSSG-SDSIWYGADRPKFLGPFS-GETPSYLNGEFAGDYGWDTAG (30-85)
Lhcbm11_Pp3c2_36380V3.1        NV--EARVTMRRTVSKSAG-SDSIWYGADRPKFLGPFS-GETPSYLTGEFAGDYGWDTAG (30-85)
Lhcbm12_Pp3c10_3020V3.1        NV--EARVTMRRTVSKSAG-SDSIWYGADRPKFLGPFS-GETPSYLNGEFAGDYGWDTAG (60-115)
Lhcbm13_Pp3c4_17240V3.1        NV--EARVSMRKSASKSAA-SDSIWYGADRPKYLGPFS-GETPSYLNGEFAGDYGWDTAG (30-85)
Lhcbm14_Pp3c3_4140V3.1         NV--DARVTMRRTVSKSAR-SDSIWYGADRPKFLGPFS-GETPSYLNGEFAGDYGWDTAG (30-85)
Lhcb1.1_AT1G29920.1            EVLGSGRVTMRKTVAKPKGPSGSPWYGSDRVKYLGPFS-GESPSYLTGEFPGDYGWDTAG (26-84)
Lhcb1.2_AT1G29910.1            EVLGSGRVTMRKTVAKPKGPSGSPWYGSDRVKYLGPFS-GESPSYLTGEFPGDYGWDTAG (26-84)
Lhcb1.3_AT1G29930.1            EVLGSGRVTMRKTVAKPKGPSGSPWYGSDRVKYLGPFS-GESPSYLTGEFPGDYGWDTAG (26-84)
Lhcb1.4_AT2G34430.1            EVFGTGRITMRK-ASKPTGPSGSPWYGSDRVKYLGPFS-GEPPSYLTGEFPGDYGWDTAG (26-83)
Lhcb1.5_AT2G34420.1            DVLGSGRVTMRKTVAKPKGPSGSPWYGSDRVKYLGPFS-GEPPSYLTGEFPGDYGWDTAG (24-82)
Lhcb2.1_AT2G05100.1            VS-GGGRVTMRRTVKSTP---QSIWYGPDRPKYLGPFS-ENTPSYLTGEYPGDYGWDTAG (29-83)
Lhcb2.2_AT2G05070.1            VL-GGGRVTMRRTVKSTP---QSIWYGPDRPKYLGPFS-ENTPSYLTGEYPGDYGWDTAG (29-83)
Lhcb2.3_AT3G27690.1            ASNGGGRIIMRRTVKSTP---QSIWYGPDRPKYLGPFS-ENTPSYLTGEYPGDYGWDTAG (29-84)
Lhcbm1_Cre01.g066917.t1.1      VKVEARRTVKPASKASTPD---SFWYGPERPLFLGAFT-GEPPSYLTGEFPGDYGWDTAG (20-75)
Lhcbm2_Cre12.g548400.t1.2      VSGRSARVVPRAA---------IEWYGPDRPKFLGPFSEGDTPAYLTGEFPGDYGWDTAG (17-67)
Lhcbm3_Cre04.g232104.t1.1      GKKGTGKTAAKQAPA-SSG---IEFYGPNRAKWLGPYSENATPAYLTGEFPGDYGWDTAG (20-75)
Lhcbm4_Cre06.g283950.t1.2      GKKTAAKAAAPKS----SG---VEFYGPNRAKWLGPYSENSTPAYLTGEFPGDYGWDTAG (20-72)
Lhcbm6_Cre06.g285250.t1.2      GKKTAAKAAAPKS----SG---VEFYGPNRAKWLGPYSENATPAYLTGEFPGDYGWDTAG (19-71)
Lhcbm7_Cre12.g548950.t1.2      VSSRSARVVPRAA---------IEWYGPDRPKFLGPFSEGDTPAYLTGEFPGDYGWDTAG (17-67)
Lhcbm8_Cre06.g284250.t1.2      GKKTAAKAAAPKS----SG---VEFYGPNRAKWLGPYSENSTPAYLTGEFPGDYGWDTAG (20-72)
Lhcbm9_Cre06.g284200.t1.2      GKKTAAKAAAPKG----AG---IEFYGPNRAKWLGPYSENSTPAYLTGEFPGDYGWDTAG (20-72)
```

Supplemental Table S1. List of identified proteins and related peptides in SDS-PAGE bands, lpBN-PAGE bands and 2D-lpBN-SDS-PAGE spots (Excel file).

In the table each tab refers to the analysis of the corresponding band for the analysis of STN7 in SDS-PAGE, thylakoid protein complexes in lpBN-PAGE and LHCB6 in 2D-lpBN-SDS-PAGE. For the proteins identified, the headers indicate: Protein FDR (false discovery rate); accession from Uniprot (see Gerotto *et al.,* 2019); protein description; percentage of coverage of the whole sequence (including transit peptide, when present); number of identified peptides (# Peptides); number of peptides spectrum matches (# PSMs); number of unique peptides (# Unique Peptides); number of protein groups the protein belongs to (# Protein Groups); length of the protein sequence as number of amino acids (#AA); molecular weight of the protein; (MW [kDa]); the isoelectric point calculated for the protein (calc. pI); Mascot protein score, which represents the sum of the scores of all peptides identified for the protein (Score Mascot); post-translational modification (protein N-terminal acetylation, Thr, Ser or Tyr phosphorylation) found: the number following the AA (e.g. T48) refers to the position of the phosphorylation/acetylation in the protein sequence (unprocessed), followed by the percentage of probability that the identified residue is the best site according to ptmRS (in brackets). Note that here only the phospho-sites prediction based on ptmRS is shown.

For each PSM of the identified peptides for each protein (second layer openable for each protein row) is indicated: Percolator q-value; Percolator PEP (posterior error probability); confidence according to Mascot score; PSM ambiguity (if the identification of the peptide was ambiguous); annotated sequence of the tryptic peptide; number and type of modification and relative position in the annotated peptide sequence; number and type of modification and relative position in the protein sequence indicated by the accession; number of identified proteins in which the peptide sequence is contained (# Protein); the accessions of the identified proteins the peptide is contained in (Protein Accessions); the number of cleavage sites within the peptide that were missed by the digestion process (# Missed Cleavages); Charge of the detected peptide; Mascot Expectation Value for strict significance (significance <0.05); rank in the list of peptide matches to the same spectrum according to the main score (Rank); rank in the list of peptide matches as assigned by search engine (search engine rank); number of identified protein groups in which the peptide sequence is contained in (# Protein groups); normalized score difference between the two top score for the peptides identified for the spectrum (DeltaScore); the normalized score distance of a higher rank peptide match to the best matching peptide (DeltaCn); m/z [Da]; observed MH+ [Da]; the theoretical MH+ mass in Dalton, calculated from the amino acid sequence, including the modifications l MH+ [Da]; the relative difference between observed and measured mass (Delta M [ppm]); the absolute difference between observed and measured mass (Delta m/z [Da]); Activation Type of the spectrum when the peptide was identified; MS order; isolation interference (in %) of the co-isolated ions within the precursor isolation window; ion inject time (in ms); first scan number in which the PSM was identified; Mascot identity threshold for strict significance (Identity Strict); Mascot identity threshold for relaxed significance (Identity Relaxed); estimate of the probability (0-100%) that the site is truly modified (ptmRS: Best Site Probabilities); the quality of the match between a modification position isoform and the respective tandem spectrum (ptmRS: Binomial Peptide Score); estimate of the

probability (0-100%) that an individual isoform is correct (ptmRS: Isoform Confidence Probability [Probability]); Sequences in Protein; the position of the peptide in the protein (Positions in Protein); PSM Modification Positions in Protein.

Supplemental Table S2. Primers used for the generation and screening of *stn7.1* and *stn7.2* knock-out (KO) plants

The table reports the primers used to generate and characterize *stn7.1* (Pp3c4_25980) and *stn7.2* (Pp3c26_5140) genes disruption in Physcomitrella KO lines.

To obtain Physcomitrella lines depleted in the expression of STN7.1 and/or STN7.2 kinase isoforms, the primers were designed to disrupt the genomic coding sequence with the insertion of a resistance cassette, Hygromycin B and Zeocin for *STN7.1* and *STN7.2*, respectively, as done previously (Alboresi et al., 2010; Gerotto et al., 2016). Genomic DNA from WT mosses was used as starting template to amplify selected homologous regions to drive the gene disruption.

Amplified *STN7.1* upstream homologous region (primers STN7.1 upstream FOR/REV) was cloned, after digestion with HindIII-XhoI endonucleases, upstream the HygB resistance cassette into BHRf plasmid; *STN7.1* downstream homologous region (primers STN7.1 downstream FOR/REV) was cloned into the same vector after digestion with SpeI-PacI restriction enzymes. Moss protoplasts were transformed after digestion of the resulting vector with HindIII-PacI.

In the case of *stn7.2*, the selected upstream homologous sequence, due to the inability to obtain an enough pure PCR product after genomic DNA amplification with primers STN7.2 upstream FOR/REV, was ordered as synthetic fragment (GeneArt). The upstream homologous region was cloned into BZRf vector (harboring zeoR cassette) after digestion with AvrII-SalI restriction enzymes and the downstream region (primers STN7.2 downstream FOR/REV) after digestion with HpaI and PacI. Moss protoplasts were transformed with the cassette linearized by digestion with AvrII-PacI.

The disruption of the target gene by homologous recombination was then verified by amplifying the flaking regions of the integrated antibiotic resistance cassette, i.e. the "Left" and "Right" Borders (LB and RB) of the integration cassette. Primer pairs to amplify LB and RB are designed so that one primer anneals in the genomic DNA external to the target homologous region used to drive the insertion, while the other anneals inside the resistance cassette. Thus, only resistant lines with the recombination event into the target locus will produce amplicons.

stn7.1 KO. After moss transformation and two round of selection, homologous recombination in HygB resistant lines was evaluated. Primers *stn7.1* LB FOR/REV and *stn7.1* RB FOR/REV allowed to amplify Left (LB) and Right Border (RB), respectively, of the integrated cassette in *stn7.1* KO lines. Primers *stn7.1* LB FOR and *stn7.1* RB REV anneal in the genomic region external to the target recombination regions, while primers *stn7.1* LB REV and *stn7.1* RB FOR anneal in the integrated resistance cassette. Finally, primers *STN7.1* RNA FOR/REV allowed to check *STN7.1* expression on cDNA by RT-PCR, producing no amplicons in *stn7.1* KO lines.

stn7.2 KO. The verification of *stn7.2* LB required a specific design. A first PCR with primers *stn7.2* LB FOR/REV (PCR I) was run, then the gel region in the size range 1.5-1.9 kb (expected size of the amplicon 1.7 kb) was cut and the eluted DNA fragment and used as template for a second PCR with primers *stn7.2* LB FOR/REV (PCR II), the result of which is shown in the Figure 1. In PCR I primer FOR anneals in the genome

external to the homologous region, in PCR II primer FOR anneals on genomic STN7.2 locus, while in both cases primer REV anneals in the resistance cassette. For the other screening PCRs, primers were designed similarly to the case of *stn7.1*.

STN8 FOR/REV, amplifying *STN8* gene (Gerotto et al., 2019), were used as PCR controls both for genomic DNA and cDNA templates.

PCR	Primer sequence	Use
STN7.1 **Upstream FOR (HindIII)**	TAACCATCAGATTAGTAGTAAAGCTTATTCTG	*stn7.1* KO generation (upstream HR)
STN7.1 **upstream REV (XhoI)**	GTCTCGAGCAACGTCTAACAGGGAGAACG	*stn7.1* KO generation (upstream HR)
STN7.1 **downstream FOR (SpeI)**	ACACTAGTCACCTGCTGGTCCGGATATTC	*stn7.1* KO generation (downstream HR)
STN7.1 **downstream REV (PacI)**	CGTTAATTAATGGTCAAGATACTTGGCACTCC	*stn7.1* KO generation (downstream HR)
stn7.1 **LB FOR**	GCTGAGAATATAATACCAAGTCAGC	*stn7.1* KO screening (LB)
stn7.1 **LB REV**	GTGTCGTGCTCCACCATGT	*stn7.1* KO screening (LB)
stn7.1 **RB FOR**	CCGCTGAAATCACCAGTCTC	*stn7.1* KO screening (RB)
stn7.1 **RB REV**	TACCCTTGCATTAAACCAAGAAAC	*stn7.1* KO screening (RB)
STN7.1 **RNA FOR**	TCGGACCAGGCAAAGGAGGTG	*stn7.1* KO screening (cDNA)
STN7.1 **RNA REV**	TGGCCTCGACTAATTTCCGCC	*stn7.1* KO screening (cDNA)
STN7.2 **Upstream FOR (AvrII)**	ATCCTAGGTGCATGTGACCCATTCCGAC	*stn7.2* KO generation (upstream HR)
STN7.2**upstream REV (SalI)**	CTGTCGACGCTGGTCATGAGCTGAATGC	*stn7.2* KO generation (upstream HR)
STN7.2 **downstream FOR (HpaI)**	CAGTTAACTTTCTGCCCTGTAATCCTCC	*stn7.2* KO generation (downstream HR)
STN7.2 **downstream REV (PacI included in amplified PCR)**	TAGGTCGTAGACTTATTTAAACTTGGAG	*stn7.2* KO generation (downstream HR)
stn7.2 **LB FOR (PCR I)**	ACCTAAACAATCGGACCAAAACG	*stn7.2* KO screening (LB)
stn7.2 **LB REV (PCR I)**	CCAGTCTTTACGGCGAGTTC	*stn7.2* KO screening (LB)
stn7.2 **LB FOR (PCR II)**	TCAGAGCGATATCAACGGGG	*stn7.2* KO screening (LB)
stn7.2 **LB REV (PCR II)**	TCTTGATGAGACCTGCTGCG	*stn7.2* KO screening (LB)
stn7.2 **RB FOR**	CGCTGAAATCACCAGTCTCTCT	*stn7.2* KO screening (RB)
stn7.2 **RB REV**	TGGGTTTGCGTTTGATCTTGATG	*stn7.2* KO screening (RB)
STN7.2 **RNA FOR**	GGGACGTAGTGTATCGGAGC	*stn7.2* KO screening (cDNA)
STN7.2**RNA REV**	ATTATAGCGGGAGGGGCTGAG	*stn7.2* KO screening (cDNA)
STN8 **FOR**	GGAAGCGGACGGATTTTGTG	PCR control
STN8 **REV**	CCCACGCAACACATTTGGAG	PCR control

Supplemental Table S3. MS confirmation of *stn7* double KO lines. List of STN7.1, STN7.2 and STN8 peptides identified in the WT and *stn7* double KO lines #1 and #2 (*stn7* dKO #1 and 2, respectively). Full data are in Supplemental Table S1. AC, protein accession from Uniprot; PSM, peptides spectrum matches.

Protein	AC	Peptide sequence	Positions in Protein	WT (PSM)	*stn7* dKO # 1 (PSM)	*stn7* dKO # 2 (PSM)
STN7.1 (A9SRW8)	A9T3J7; A9SRW8	[K].DGPLVVK.[RK]	[191-197]	1	nd	nd
	**A9SRW8**	**[K].DGPLVVKK.[A]**	**[191-198]**	1	nd	nd
	**A9SRW8**	**[R].DFPYNVEELLLGPGK.[G]**	**[260-274]**	1	nd	nd
	**A9SRW8**	**[R].QILSALAQLHSTGIVHR.[D]**	**[295-311]**	2	nd	nd
	**A9SRW8**	**[R].DIKPQNIIYSEETK.[S]**	**[312-325]**	1	nd	nd
	A9T3J7; A9SRW8	[K].IIDLGAAADLR.[V]	[329-339]	1	nd	nd
	A9T3J7; A9SRW8	[R].VGINYIPK.[E]	[340-347]	1	nd	nd
	A9T3J7; A9SRW8	[K].EFLLDPR.[Y]	[348-354]	1	nd	nd
	**A9SRW8**	**[R].SDSGLISFNR.[Q]**	**[411-420]**	1	nd	nd
	**A9SRW8**	**[R].ISANAALAHPYFSR.[E]**	**[474-487]**	1	nd	nd
	**A9SRW8**	**[R].EGLLGLSIMQR.[L]**	**[488-498]**	1	nd	nd
	**A9SRW8**	**[R].GAQPDNEGVDWVTSLMAK.[S]**	**[506-523]**	2	nd	nd
	A9T3J7; A9SRW8	[R].RNAmAQALR.[LI]	[553-561]	1	nd	nd
	A9T3J7; A9SRW8	[R].NAMAQALR.[LI]	[554-561]	2	nd	nd
**Tot STN7.1 PSM**				**17**	**0**	**0**
STN7.2 (A9T3J7)	**A9T3J7**	**[K].KLGEGAFGTVFK.[A]**	**[167-178]**	1	nd	nd
	A9T3J7; A9SRW8	[K].DGPLVVK.[RK]	[190-196]	1	nd	nd
	**A9T3J7**	**[K].DGPLVVKR.[A]**	**[190-197]**	1	nd	nd
	**A9T3J7**	**[K].GKEEFWLLWR.[Y]**	**[235-244]**	1	nd	nd
	**A9T3J7**	**[R].KGEDLPR.[G]**	**[273-279]**	1	nd	nd
	A9T3J7; A9SRW8	[K].IIDLGAAADLR.[V]	[328-338]	1	nd	nd
	A9T3J7; A9SRW8	[R].VGINYIPK.[E]	[339-346]	1	nd	nd
	A9T3J7; A9SRW8	[K].EFLLDPR.[Y]	[347-353]	1	nd	nd
	**A9T3J7**	**[K].RRDYDLVK.[W]**	**[423-430]**	1	nd	nd
	**A9T3J7**	**[R].ISANAALAHPYFER.[A]**	**[473-486]**	1	nd	nd
	**A9T3J7**	**[K].SGTDSVGGFTEAQLQNIR.[E]**	**[523-540]**	1	nd	nd
	A9T3J7; A9SRW8	[R].RNAmAQALR.[LI]	[552-560]	1	nd	nd
	A9T3J7; A9SRW8	[R].NAMAQALR.[LI]	[553-560]	2	nd	nd
**Tot STN7.2 (A9T3J7) PSM**				**14**	**0**	**0**
STN8 (A9RZZ4)	A9RZZ4	[K].SLVELSDFER.[Y]	[131-140]	1	1	1
	A9RZZ4	[R].AAGALGIGFIYLTAKPGVLK.[G]	[143-162]	1	1	1
	A9RZZ4	[K].GAFDMYIGAPAQAAIENLR.[G]	[163-181]	1	nd	Nd
	A9RZZ4	[K].RTDFVIDQR.[L]	[188-196]	nd	nd	1
	A9RZZ4	[R].RLEEFEDYKK.[F]	[227-236]	1	1	1
	A9RZZ4	[R].TLNNDDEIKR.[N]	[328-337]	1	1	1
	A9RZZ4	[R].NSLIITQIMR.[Q]	[338-347]	1	nd	Nd
	A9RZZ4	[R].DVKPSNVVVTDKGK.[L]	[365-378]	1	1	nd
	A9RZZ4	[K].FIDFGAATDLR.[V]	[381-391]	1	1	1
	A9RZZ4	[R].TSIGLQTFKK.[E]	[463-472]	nd	nd	1
	A9RZZ4	[R].INFDLLDADGGKGWDLATK.[L]	[493-511]	1	Nd	Nd
	A9RZZ4	[R].LSAESALR.[H]	[524-531]	1	Nd	nd
**Tot STN8 (A9RZZ4) PSM**				**10**	**6**	**7**

Supplemental Table S4. Chlorophyll content after long-term acclimation. Chl *a/b* ratios of the WT and *stn7* double KO lines #1 and #2 (*stn7* double KO #1 and 2, respectively) after growth in CL (45 µmol photons m^{-2} s^{-1}) conditions 11 days, or moving 4-days old plates to LL, FL or HL (7, 25/800 or 600 µmol photons m^{-2} s^{-1}, respectively) for one week. Data are shown as average ± SD (n≥3).

	**WT**	***stn7* double KO #1**	***stn7* double KO #2**
**CL**	2.54 ± 0.05	2.39 ± 0.05	2.37 ± 0.05
**Long-LL**	2.56 ± 0.04	2.32 ± 0.10	2.43 ± 0.05
**Long-FL**	2.54 ± 0.04	2.43 ± 0.09	2.33 ± 0.15
**Long-HL**	2.65 ± 0.13	2.82 ± 0.16	2.76 ± 0.16
